# TAZ deficiency impairs the autophagy-lysosomal pathway through NRF2 dysregulation and lysosomal dysfunction

**DOI:** 10.7150/ijbs.88897

**Published:** 2024-04-22

**Authors:** Hyo Kyeong Kim, Hana Jeong, Mi Gyeong Jeong, Hee Yeon Won, Gibbeum Lee, Soo Han Bae, Miso Nam, Sung Hoon Lee, Geum-Sook Hwang, Eun Sook Hwang

**Affiliations:** 1 College of Pharmacy and Graduate School of Pharmaceutical Sciences, Ewha Womans University, Seoul 03760, Korea.; 2 College of Medicine, Severance Biomedical Science Institute, Yonsei University, Seoul 03722, Korea.; 3 Integrated Metabolomics Research Group, Western Seoul Center, Korea Basic Science Institute, Seoul 03759, Korea.; 4 College of Pharmacy, Chung-Ang University, Seoul 06974, Korea.

**Keywords:** autophagy, lysosomal acidification, NRF2, oxidative stress, TAZ

## Abstract

Transcriptional coactivator with a PDZ-binding motif (TAZ) plays a key role in normal tissue homeostasis and tumorigenesis through interaction with several transcription factors. In particular, TAZ deficiency causes abnormal alveolarization and emphysema, and persistent TAZ overexpression contributes to lung cancer and pulmonary fibrosis, suggesting the possibility of a complex mechanism of TAZ function. Recent studies suggest that nuclear factor erythroid 2-related factor 2 (NRF2), an antioxidant defense system, induces TAZ expression during tumorigenesis and that TAZ also activates the NRF2-mediated antioxidant pathway. We thus thought to elucidate the cross-regulation of TAZ and NRF2 and the underlying molecular mechanisms and functions. TAZ directly interacted with NRF2 through the N-terminal domain and suppressed the transcriptional activity of NRF2 by preventing NRF2 from binding to DNA. In addition, the return of NRF2 to basal levels after signaling was inhibited in TAZ deficiency, resulting in sustained nuclear NRF2 levels and aberrantly increased expression of NRF2 targets. TAZ deficiency failed to modulate optimal NRF2 signaling and concomitantly impaired lysosomal acidification and lysosomal enzyme function, accumulating the abnormal autophagy vesicles and reactive oxygen species and causing protein oxidation and cellular damage in the lungs. TAZ restoration to TAZ deficiency normalized dysregulated NRF2 signaling and aberrant lysosomal function and triggered the normal autophagy-lysosomal pathway. Therefore, TAZ is indispensable for the optimal regulation of NRF2-mediated autophagy-lysosomal pathways and for preventing pulmonary damage caused by oxidative stress and oxidized proteins.

## Introduction

Transcriptional co-activator with a PDZ-binding motif (TAZ) is an important mediator of the Hippo pathway that regulates organ development and size control 1,2. Based on multiple protein-protein interactive domains, TAZ interacts with several transcription factors, including the TEAD family, and regulates their DNA-binding and transcriptional activity, thereby modulating the development and function of lung, bone, cartilage, adipose, muscle, and testis 3-9. TAZ also contributes to liver regeneration, colonic epithelium rejuvenation, and alveolar regeneration in response to tissue damage, and its deficiency causes emphysematous changes in lung tissue and damage-induced liver fibrosis 10-17. TAZ has also been shown to increase insulin sensitivity via induction of muscle IRS1 and promote exercise-induced muscle regeneration and adaptation via mitochondrial biogenesis 18-20. Despite the importance of TAZ in the homeostatic control of normal tissues 21, TAZ activation is prominent in tumors and plays an important role in tumor progression and metastasis 22. TAZ promotes tumor cell migration and invasion and induces chemoresistance and immune evasion, exacerbating tumor prognosis 23-25. Notably, TAZ is pervasively upregulated in human patients with lung cancer and idiopathic pulmonary fibrosis and contributes to disease severity in the pulmonary system 26,27. Consequently, TAZ inhibition has been proposed as a beneficial therapeutic approach for anti-tumor and anti-fibrosis therapy. Due to the complexity of TAZ function in normal lung and lung cancer, it would be important and interesting to elucidate the correlation of TAZ with signaling pathways leading to homeostatic control or tumorigenesis.

The nuclear factor erythroid 2-related factor 2 (NRF2) signaling pathway is a prominent defense system that protects lung epithelial cells against oxidative stress by activating antioxidants, lysosomal biogenesis, and autophagy pathways; however, NRF2 hyperactivation has been implicated in lung cancer development and metastasis, with known functional complexity 28-30. NRF2 is maintained at low levels without cellular stress through proteasomal degradation and is inactivated by interacting with Kelch-like ECH-associated protein 1 (KEAP1) 31,32. However, reactive oxygen species (ROS) or electrophilic sulforaphane (SFN) induce the expression and nuclear localization of NRF2 31,33,34. Nuclear NRF2 directly binds to the antioxidant-responsive element (ARE) within the gene promoter and increases the expression of antioxidants, including sulfiredoxin (SRX), glutathione S-transferase A (GSTA), heme oxygenase-1 (HMOX1), NAD(P)H quinone oxidoreductase 1 (NQO1), and catalase (CAT), and autophagy receptor p62/SQSTM1 (sequestosome 1, hereafter referred to as p62), thereby reducing intracellular oxidative stress and protecting against cell damage through the autophagy pathway 35,36. Very recently, it was reported that NRF2 stimulates the activity of transcription factor EB (TFEB) to promote lysosomal biogenesis, indicating the importance of NRF2 activity in regulating the autophagy-lysosomal degradation pathway 37. However, hyperactivation of NRF2 led to the overexpression of p62, resulting in dysregulation of autophagy, and dysregulated p62 accumulation prolonged NRF2 activation, contributing to the pathological exacerbation of lung cancer and liver diseases 38-41. There is growing evidence that dysregulation of autophagy, such as persistent or insufficient autophagy, can be detrimental to cells and tissues and lead to various human diseases 42-45. Moreover, persistent activation of NRF2 due to the defect of KEAP1 promotes tumor growth, metastasis, and chemoresistance and leads to poor clinical outcomes 29,46-49. Therefore, optimal and timely regulation of the NRF2 signaling pathway is essential to eliminate intracellular ROS and oxidized proteins and prevent pathogenic progression.

Recent studies suggest that NRF2 increases the expression of TAZ and promotes tumorigenesis, and TAZ also increases and activates NRF2 to alleviate oxidative damage in uterine decidualization and neuroinflammation 29,50-52. In this study, we aimed to investigate whether and how TAZ is linked to the homeostatic control of the NRF2 signaling pathway in the pulmonary system.

## Results

### TAZ physically interacts with NRF2 *in vitro* and *in vivo*

**Figure 1:**
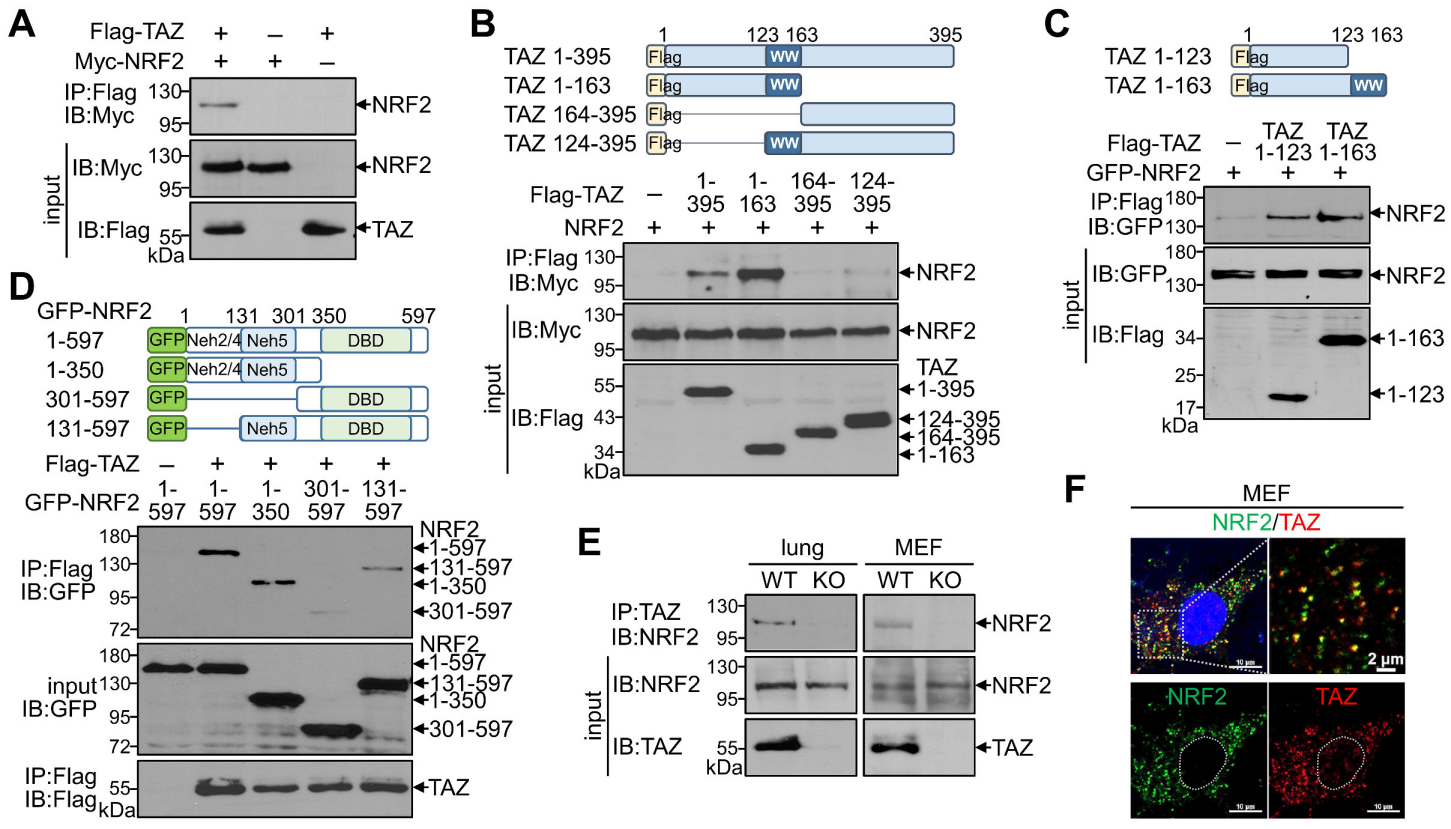
** Physical interaction of TAZ with NRF2.** (**A-D**) Highly transfectable HEK-293T cells were transfected with expression vectors of different forms of TAZ and NRF2 and subjected to a protein-protein interaction assay. Flag-tagged full-length TAZ and Myc-linked NRF2 were overexpressed, and protein complexes precipitated with Flag antibody were analyzed with Myc antibody (**A**). The schematic structure of TAZ truncations is shown. The Protein-protein interaction between TAZ truncations and NRF2 was analyzed (**B**). Protein-protein interaction assay of TAZ truncations (TAZ 1-123 and TAZ 1-163) with NRF2 (**C**). Schematic structure of NRF2 truncations. Protein-protein interaction assay between NRF2 truncations and TAZ (**D**). (**E**) Protein-protein interaction assay of endogenous TAZ and NRF2 in mouse lung and MEF cells from WT and TAZ KO mice. Endogenous TAZ protein complexes were precipitated and analyzed with an NRF2-specific antibody. (**F**) IF staining of endogenous TAZ and NRF2 in MEF cells. All experiments were conducted at least three times, and representative images are shown.

Given the structural characterization of TAZ with multiple protein-protein interaction domains, we first sought to determine whether TAZ interacts with NRF2. Protein-protein interactions using overexpressed TAZ and NRF2 confirmed a direct physical interaction between TAZ and NRF2 (Fig. 1A). Subsequent interaction assays with truncated proteins revealed that the N-terminal domain of TAZ (TAZ 1-163) interacted with NRF2, whereas the C-terminal domain of TAZ containing the WW domain (TAZ 124-395) did not interact with NRF2 (Fig. 1B). Interestingly, the N-terminal 123-amino acid (aa) region of TAZ (TAZ 1-123), but not the WW domain, was sufficient and critical for binding to NRF2 (Fig. 1C). Further analysis using NRF2 truncations revealed that the N-terminal domain of NRF2 (NRF2 1-350) was required for binding to TAZ, and NRF2 131-597 with the 1-130 aa region removed was also reduced but still bound to TAZ (Fig. 1D), suggesting that the ECH homology (Neh) domains (Neh2, Neh4, and Neh5 within the 1-300 aa region) in NRF2 are responsible for the interaction with TAZ. Endogenous TAZ was physically associated with endogenous NRF2 in mouse lung tissue and MEF cells, validating the *in vivo* TAZ-NRF2 interaction (Fig. 1E). Immunofluorescence (IF) staining revealed that endogenous TAZ colocalized with NRF2 in the cytoplasm of MEF cells without stimulation (Fig. 1F).

### TAZ suppresses NRF2 transcriptional activity in an interaction-dependent manner

**Figure 2:**
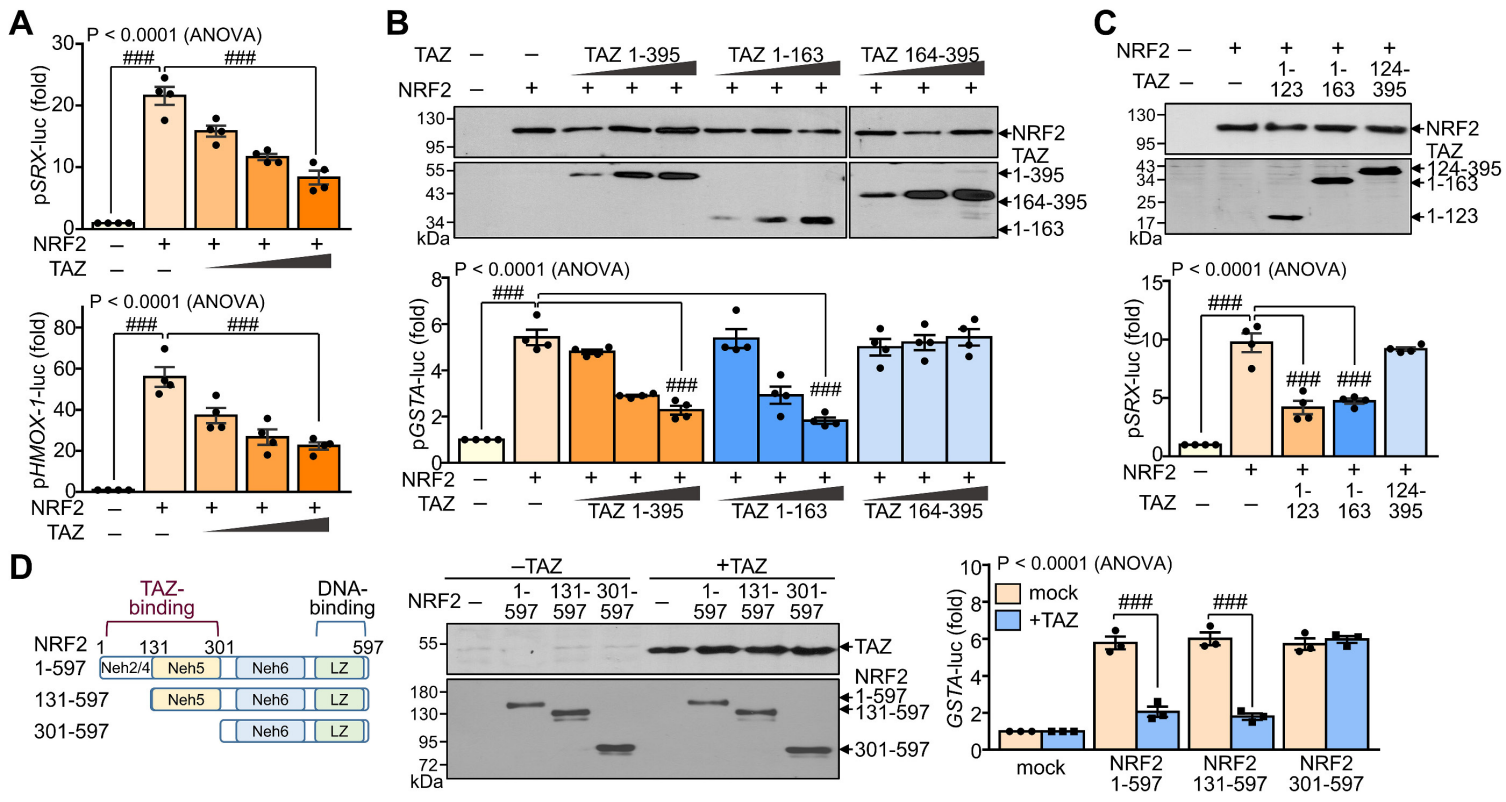
** Suppression of NRF2 transcriptional activity by TAZ.** HEK-293T cells were transfected with expression vectors of various truncations of TAZ and NRF2 with or without the reporter genes (p*SRX*-luc, p*HMOX1*-luc, or p*GSTA*-luc) and harvested for reporter gene assay. (**A**) The promoter activity of the p*SRX*-luc and p*HMOX1*-luc reporter genes was assayed by exogenous expression of NRF2 and TAZ. (**B**) Expression of TAZ truncations and reporter assay of the *GSTA* promoter activity by exogenous NRF2 and TAZ truncations. (**C**). The NRF2-induced *SRX* promoter activity was analyzed by the overexpression of TAZ truncations. (**D**) Schematic structure and expression of NRF2 truncations. The *GSTA* promoter activity was assayed by the overexpression of NRF2 truncations and TAZ. Data are given as mean ± SEM of three or four independent experiments. ###, P< 0.0001 by ANOVA with Tukey's HSD post-hoc test.

To evaluate the effect of TAZ-NRF2 interaction on the transcriptional activity of NRF2, we introduced reporter genes containing the ARE promoter, such as p*SRX*-luc, p*HMOX1*-luc, and p*GSTA*-luc, and expression vectors of NRF2 and TAZ into HEK-293T cells and determined the activity of the reporter genes. Ectopic overexpression of NRF2 markedly increased the ARE promoter activity of SRX and HMOX1 genes, which was dose-dependently decreased by forced TAZ expression (Fig. 2A). The full-length (TAZ 1-395) or N-terminal domain (TAZ 1-163) of TAZ significantly inhibited NRF2-induced ARE promoter activity, whereas the C-terminal domain of TAZ (TAZ 164-395) failed to inhibit the activity despite the similar expression level of TAZ proteins (Fig. 2B). Notably, the NRF2-binding N-terminal 123 aa region of TAZ (TAZ 1-123) was sufficient to inhibit NRF2-mediated ARE promoter activity (Fig. 2C). Furthermore, TAZ-mediated NRF2 suppression was impaired in a truncated NRF2 (NRF2 301-597), which does not contain the TAZ-binding domain (Fig. 2D), suggesting that the suppression of NRF2 activity by TAZ is dependent on their interaction.

### TAZ inhibits the DNA-binding activity of NRF2

**Figure 3:**
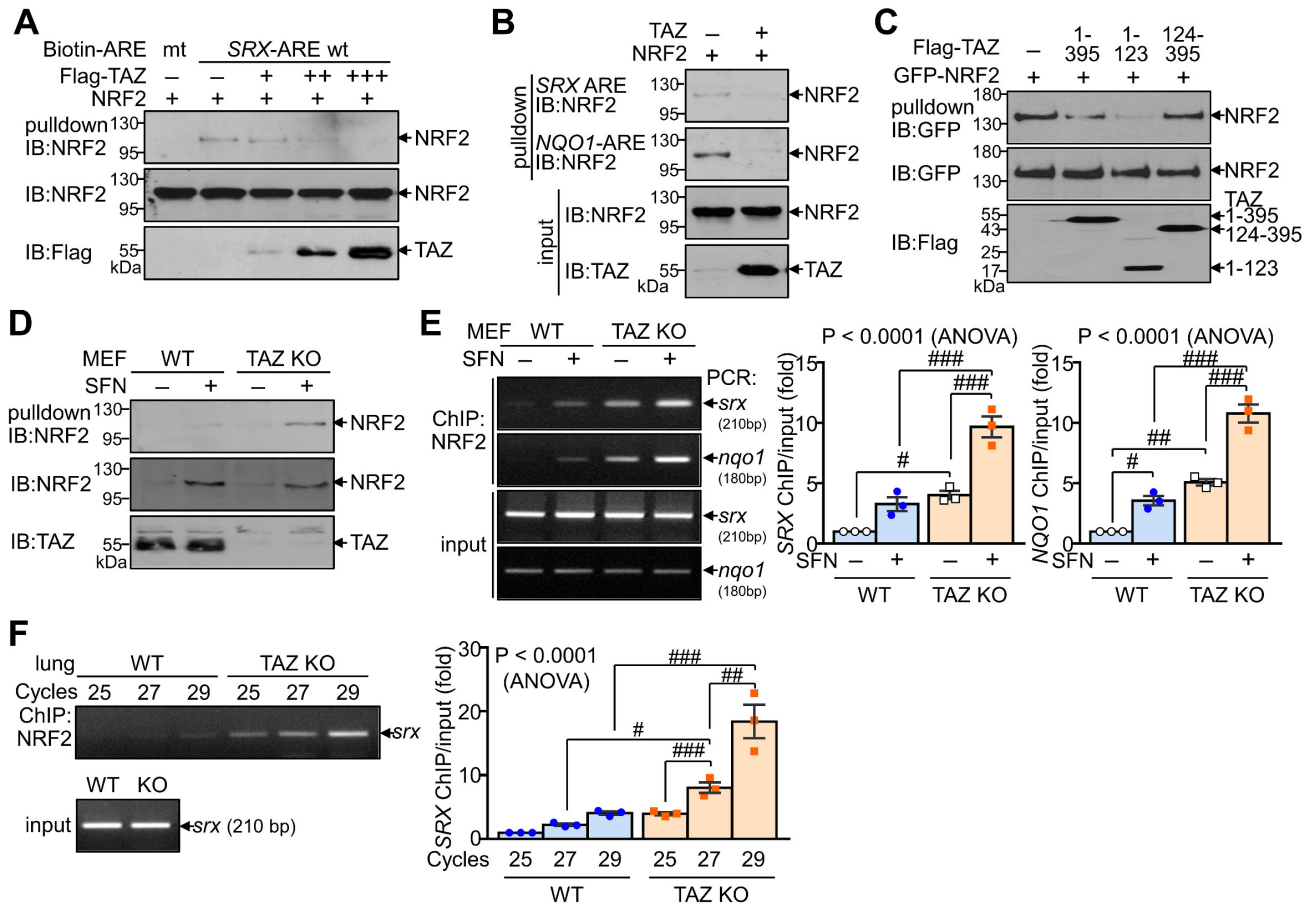
** Inhibition of DNA-binding activity of NRF2 by TAZ.** (**A**-**C**) HEK-293T cells were transfected with NRF2 and TAZ expression vector and harvested for DNA-pulldown assay. Protein extracts were incubated with biotin-labeled ARE DNA and subjected to IB analysis. DNA binding assay of NRF2 to the wild-type (wt) and mutant (mt) ARE DNA with dose-dependent TAZ expression (**A**). Analysis of NRF2 binding to the *SRX*-ARE or *NQO1*-ARE DNA in the presence or absence of TAZ (**B**). DNA binding assay of NRF2 in the presence of different TAZ truncations (**C**). (**D-E**) WT and TAZ KO MEF cells were incubated with either vehicle or SFN (10 μM) for 24 h to induce NRF2 expression and subjected to DNA pulldown assay (**D**) or ChIP and qPCR assay (**E**). (**F**) Lungs of WT and TAZ KO mice were harvested for ChIP. NRF2 binding to the *SRX* gene was analyzed by semiquantitative PCR with different cycles. Representative images of at least three independent experiments are presented. Data are given as mean ± SEM of three independent experiments. #P < 0.05; ##P < 0.0005; ###P < 0.0001 by ANOVA with Tukey's HSD post-hoc test.

We next investigated whether TAZ directly modulates the DNA-binding activity of NRF2, which is essential for regulating transcriptional activity. DNA pulldown assay showed that ectopically expressed NRF2 bound to the consensus ARE but not to the mutated ARE. Overexpression of TAZ decreased the DNA-binding activity of NRF2 in a dose-dependent manner (Fig. 3A). TAZ also dramatically reduced NRF2 binding to the *SRX*-ARE and the *NQO1*-ARE (Fig. 3B). In addition, TAZ 1-123, which is crucial for NRF2 binding and repression, was sufficient to inhibit NRF2 binding to DNA (Fig. 3C). The effect of TAZ deficiency on the DNA-binding activity of endogenous NRF2 was assessed by treating wild-type (WT) and TAZ KO MEF cells with SFN to increase the expression levels of endogenous NRF2. Comparable amounts of endogenous NRF2 from WT and TAZ KO cells were then incubated with biotinylated SRX-ARE DNA. NRF2 binding to SRX-ARE was significantly increased in TAZ KO MEF cells compared to WT cells (Fig. 3D). Chromatin immunoprecipitation (ChIP) and subsequent quantitative analysis further demonstrated that TAZ deficiency increased the binding of endogenous NRF2 to the *SRX* or *NQO1* gene promoter in MEF cells with or without SFN treatment (Fig. 3E). Semi-quantitative and quantitative real-time PCR analysis of the ChIP complex also confirmed that NRF2 binding to chromatin was significantly increased in the lungs of TAZ KO mice compared to the WT control (Fig. 3F). These results suggest a critical role for TAZ in controlling the DNA-binding activity of NRF2 both *in vitro* and *in vivo*.

### TAZ is mandatory for the progressive deactivation of activated NRF2

**Figure 4:**
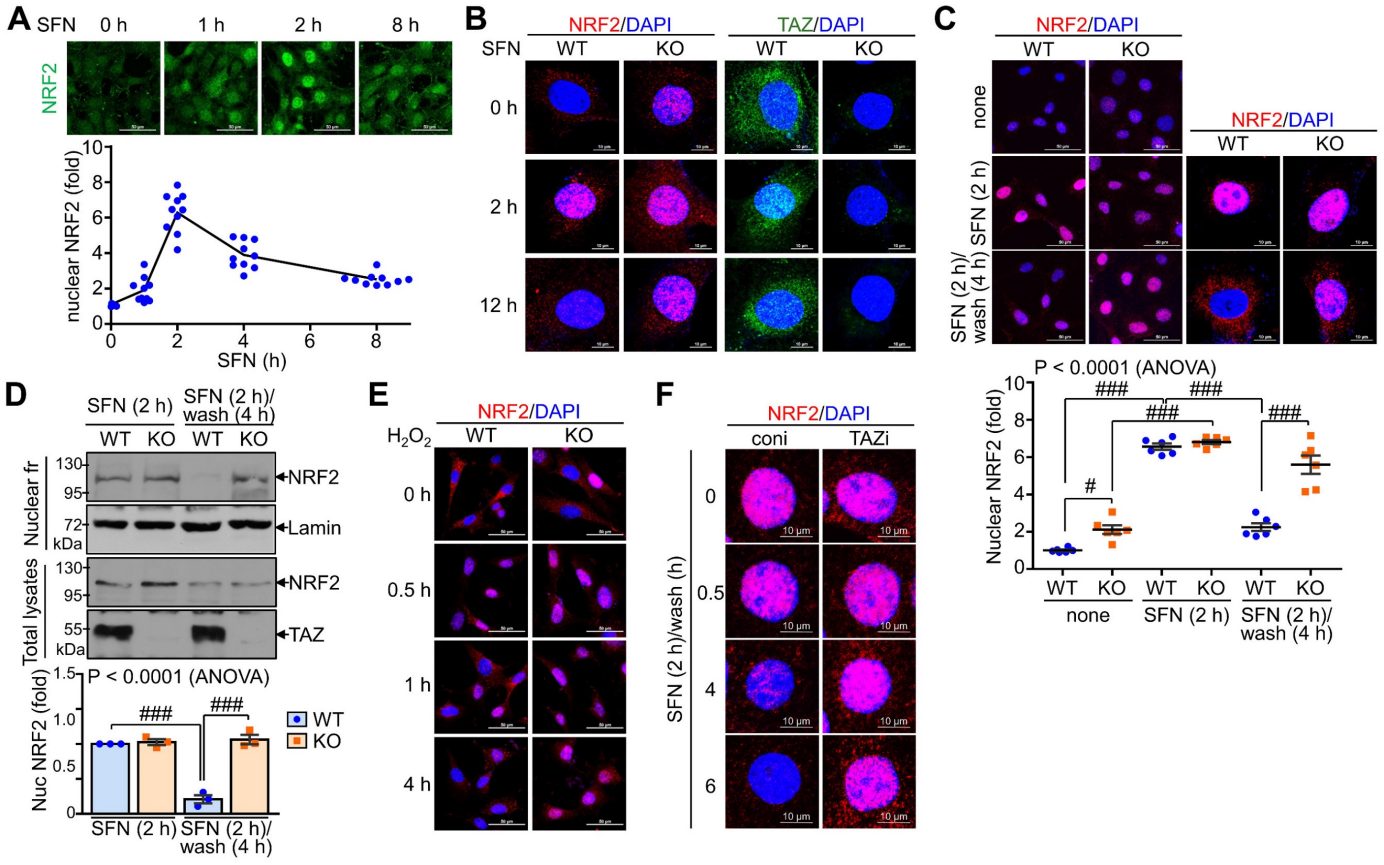
** Persistent nuclear expression of NRF2 in TAZ deficiency.** WT and TAZ KO MEF cells were incubated with SFN (10 μM) for the indicated time points and subjected to IF staining or IB analysis. (**A**) WT MEF cells were treated with SFN and harvested at different time points. Nuclear NRF2 was quantified in MEF cells in response to SFN stimulation. Ten different cell images were obtained and analyzed for nuclear NRF2 quantification. (**B**) WT and TAZ KO MEF cells were treated with SFN for 2 h and 12 h and subjected to IF staining for NRF2 and TAZ. (**C-D**) MEF cells were either treated with SFN for 2 h or with SFN for 2 h and cultured for 4 h after washout. Nuclear localization of NRF2 was analyzed by IF staining and subsequent quantitative analysis (**C**). Cells were harvested and subjected to protein extraction of nuclear fraction and total lysates. Protein extracts were then analyzed by IB analysis of NRF2 and TAZ. Lamin was used as a control for nuclear protein (**D**). Data are given as mean ± SEM. #P < 0.05; ###P < 0.0001 by ANOVA with Tukey's HSD post-hoc test. (**E**) MEF cells were treated with hydrogen peroxide (100 μM) and harvested for NRF2 staining at the indicated time points. (**F**) Control (coni) and TAZ-knockdown (TAZi) epithelial cells were stimulated with SFN for 2 h, washed with PBS, harvested at the indicated time points, and then stained for NRF2. Representative images of at least three independent experiments are presented.

We also examined the effect of TAZ on the subcellular localization of NRF2 following external stimuli such as SFN. NRF2 expression was increased by SFN stimulation, with nuclear NRF2 levels peaking at 2 hours and returning to basal levels at 8 h (Fig. 4A). NRF2 expression increased at 2 hours after SFN stimulation and then decreased, at which time TAZ also translocated to the nucleus and then to the cytoplasm. Interestingly, at 12 hours of SFN treatment, nuclear NRF2 expression remained high in TAZ KO cells, in contrast to the decrease in WT cells (Fig. 4B). Furthermore, SFN-induced nuclear NRF2 expression decreased after SFN washout in WT cells, whereas nuclear NRF2 expression persisted in TAZ KO cells (Fig. 4C). IB analysis of the nuclear fraction further confirmed the persistence of nuclear NRF2 levels in KO cells after SFN treatment and washout (Fig. 4D). Furthermore, treatment of cells with hydrogen peroxide induced nuclear localization of NRF2 in the early stages of oxidative stress with no difference between WT and KO cells. However, the cytoplasmic localization of NRF2 or its subsequent decrease in nuclear levels observed in WT cells was impaired in TAZ KO cells (Fig. 4E). Sustained nuclear expression of NRF2 following TAZ deficiency was further confirmed in TAZ knockdown epithelial cells treated with SFN and washed out (Fig. 4F), suggesting an essential role of TAZ in the progressive deactivation of activated NRF2 in the nucleus upon cellular stress.

### TAZ deficiency causes an increase in NRF2-mediated antioxidants *in vitro/in vivo*

**Figure 5:**
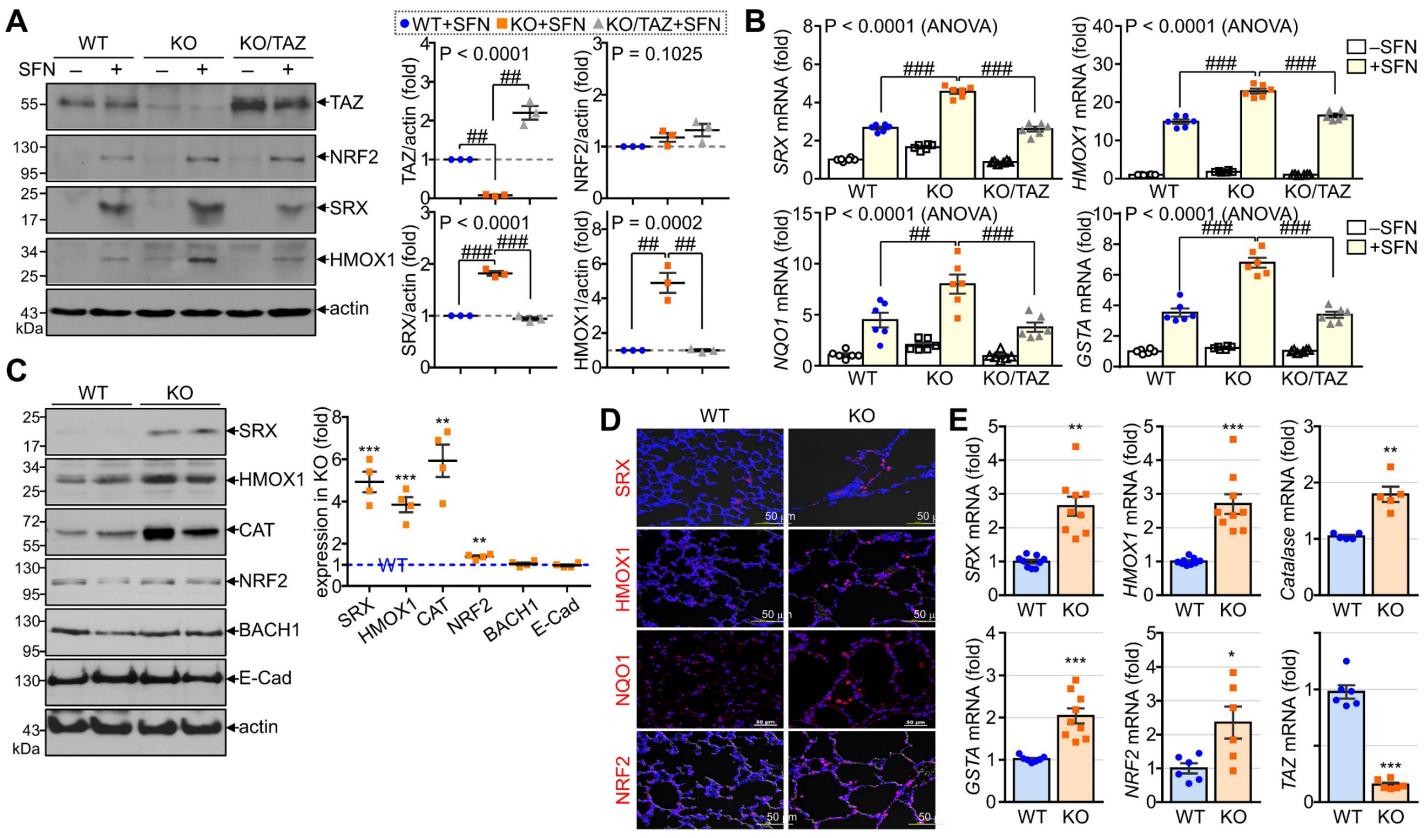
** TAZ-mediated modulation of NRF2-induced antioxidants**. (**A-B**) WT, TAZ KO, and KO/TAZ MEF cells were stimulated with either vehicle or SFN (10 μM) for 24 h, followed by protein extraction and RNA preparation. Protein lysates were resolved and analyzed by IB. Protein levels of TAZ, NRF2, SRX, and HMOX1 were determined by normalization to actin levels and expressed as fold induction compared to WT control (**A**). Total RNA was prepared and subjected to RT and qPCR analysis (**B**). ##P < 0.005; ###P < 0.0001 by ANOVA with Tukey's HSD post-hoc test. (**C-E**) Lung tissues were harvested from WT and TAZ KO mice (n = 9 per group). Protein lysates were extracted from the lung tissues and analyzed by IB and quantitative analysis. Relative expression levels of antioxidants in KO were expressed as fold change compared to WT control (**C**). Lung tissue sections were prepared and stained with antibodies against SRX, HMOX1, NQO1, and NRF2 (**D**). Total RNA was prepared and analyzed by RT and qPCR. Relative transcript levels were quantified by fold induction relative to the WT control (**E**). Representative images from at least three independent experiments are shown, and data are given as mean ± SEM. *P < 0.05; **P < 0.005; ***P < 0.0005 by two-tailed Student's t-test.

To validate the *in vitro* and *in vivo* significance of NRF2 regulation by TAZ, we first analyzed the expression levels of NRF2-induced antioxidants in TAZ-deficient MEF cells and TAZ KO lungs. To induce endogenous NRF2 expression and activation, WT, TAZ KO, and TAZ-restored KO (KO/TAZ) MEF cells were stimulated with SFN. Similar levels of NRF2 were induced in WT and TAZ KO cells by SFN stimulation, but protein levels of NRF2-driven SRX and HMOX1 were increased in TAZ KO cells compared to WT cells (Fig. 5A). SFN treatment also significantly increased the transcript levels of SRX, HMOX1, NQO1, and GSTA in WT cells and to a greater extent in TAZ KO cells (Fig. 5B). However, reintroduction of TAZ in KO cells restored the protein and transcript levels of the antioxidants to the levels observed in WT cells (Fig. 5A and B). Consistent with the *in vitro* results, SRX, HMOX1, CAT, and NRF2 were significantly upregulated in TAZ KO lungs compared to the WT control, whereas BACH1 and E-cadherin were unaffected by TAZ deficiency (Fig. 5C). IF staining showed that TAZ deficiency increased the expression of SRX, HMOX1, NQO1, and NRF2 in lungs with airway destruction (Fig. 5D). Increased antioxidants in TAZ KO lungs were evidenced at the transcriptional level (Fig. 5E), highlighting the direct function of TAZ in the transcriptional regulation of NRF2-mediated antioxidants.

### Sustained NRF2 activation in TAZ deficiency promotes p62-mediated autophagy

**Figure 6:**
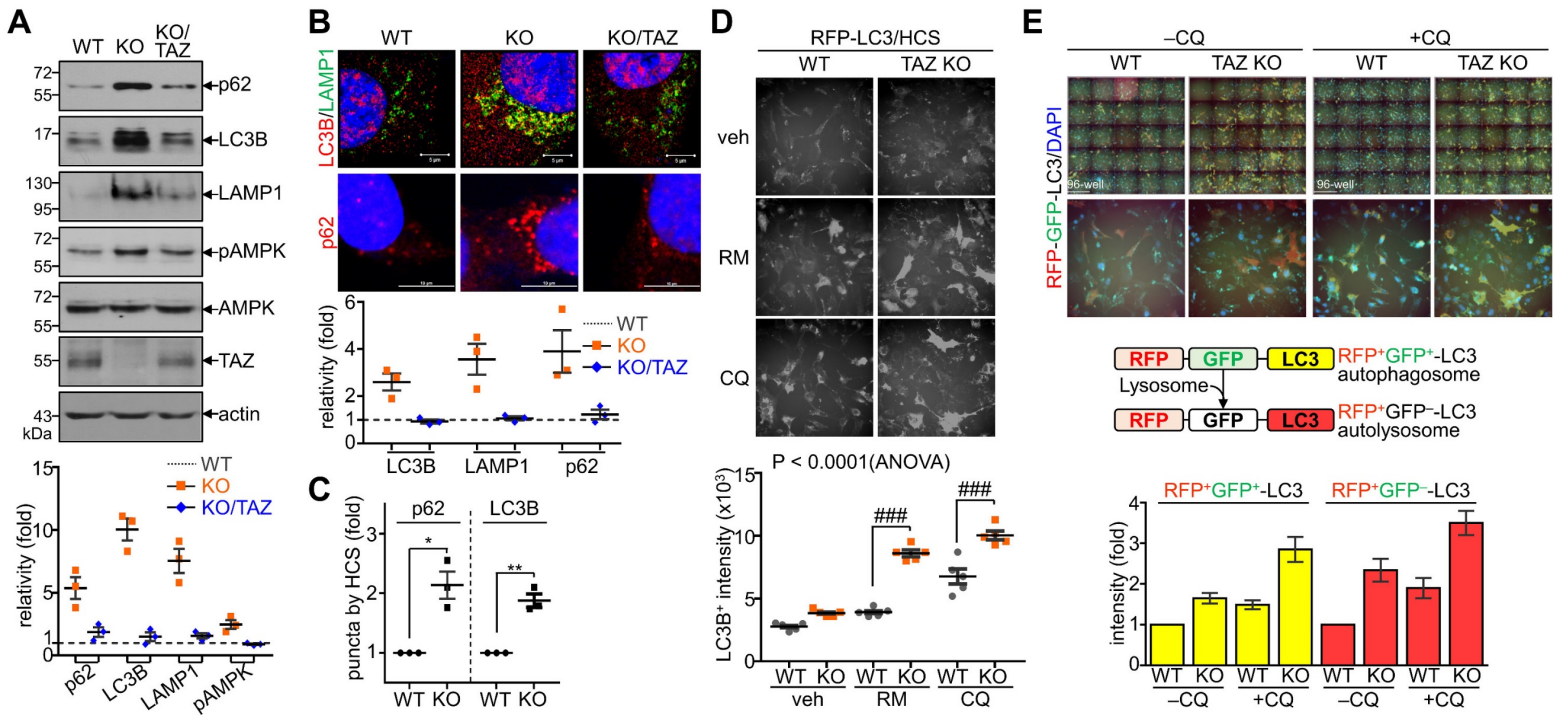

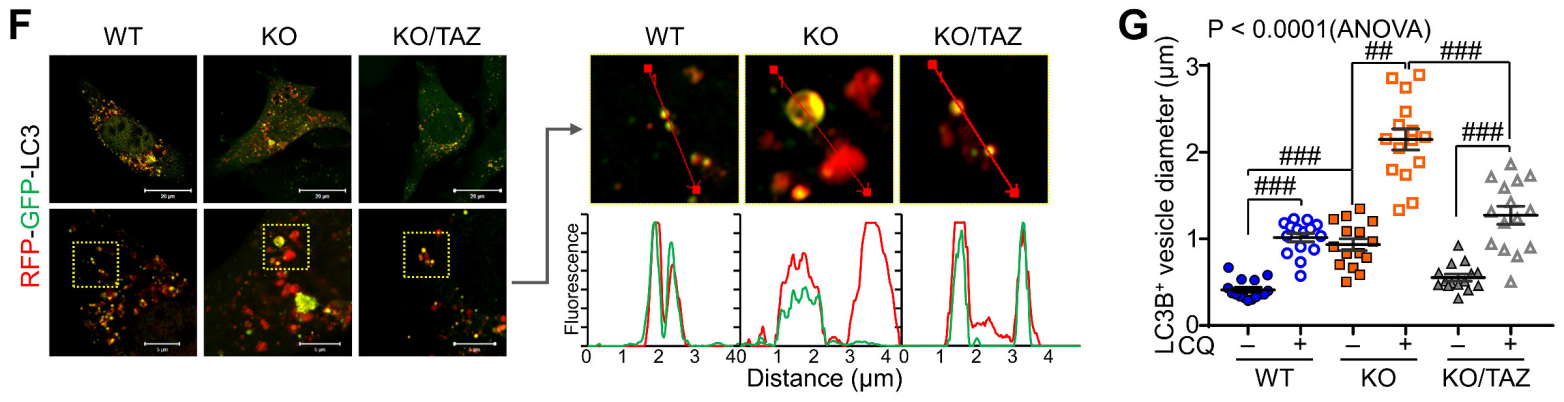
** Dysregulation of the NRF2-mediated autophagy pathway in TAZ deficiency.** (**A-C**) WT, TAZ KO, and KO/TAZ MEF cells were cultured under glucose-free conditions for 24 h and subjected to further analysis. IB analysis of autophagy-related molecules (**A**). IF staining of LC3B, LAMP1, and p62 (**B**). IF staining of p62 and LC3B followed by quantitative analysis using the HCS platform. Expression level was normalized to WT control and expressed as fold change (**C**). *P < 0.05; **P < 0.005 by two-tailed Student's t-test. (**D**) WT and KO MEF cells were transfected with RFP-LC3 vector and further incubated with either autophagy inducer rapamycin (RM, 500 nM) or CQ (50 μM) for 4 h under glucose-free conditions, followed by HCS analysis. ###P < 0.0001 by ANOVA with Tukey's HSD post-hoc test. (**E**) WT and KO MEF cells were transfected with RFP-GFP-LC3 vector and treated with either vehicle or CQ (50 μM) for 4 h, followed by HCS analysis. LC3^+^ fluorescent images obtained with the HCS platform. Fluorescence values of autophagosomes and autolysosomes were expressed as the intensity ratio of red LC3^+^ puncta (autolysosome) or yellow LC3^+^ puncta (autophagosome) in TAZ KO cells to the intensity in WT control. (**F-G**) WT, KO, and KO/TAZ MEF cells were transfected with RFP-GFP-LC3 vector and observed under a fluorescence confocal microscope. Fluorescence values of LC3^+^ puncta were analyzed with a distance (**F**). Diameters of LC3^+^ puncta were determined quantitatively (**G**). Representative images from at least three independent experiments are shown. Data are expressed as mean ± SEM. ##P < 0.005; ###P < 0.0001 by ANOVA with Tukey's HSD post-hoc test.

Next, we sought to analyze the effects of TAZ on autophagy and lysosomal biogenesis regulated by NRF2 35,37. Under glucose-deprived conditions, the expression of p62, LC3B, and lysosome-associated membrane protein 1 (LAMP1), which are involved in the autophagy-lysosomal pathway, was increased in TAZ KO cells and decreased in KO/TAZ cells. In addition, phosphorylated AMPK was also increased in TAZ KO cells and returned to basal levels upon TAZ restoration (Fig. 6A). IF staining also showed that the expression of LC3B, LAMP1, and p62 was increased in TAZ KO cells but decreased in KO/TAZ cells (Fig. 6B). Image-based high content screening (HCS) further confirmed the quantitative increase of p62 and LC3B-positive puncta in KO cells compared to WT cells (Fig. 6C), suggesting that autophagy was likely promoted in TAZ deficiency. To analyze the effect of TAZ on autophagosome maturation in the autophagy pathway, RFP-LC3 or GFP-RFP-LC3 was introduced into WT and TAZ KO MEF cells and HCS analysis was performed. Similar levels of LC3B puncta in WT and TAZ KO MEF cells were increased by treatment with the autophagy inducer rapamycin or the lysosomal inhibitor chloroquine (CQ), and especially greater in KO cells (Fig. 6D). In addition, autophagy vesicles, including autophagosomes (yellow puncta) and autolysosomes (red puncta), were significantly increased in TAZ KO cells. LC3B-positive autolysosomes were abnormally enlarged in TAZ KO cells, and autophagosomes accumulated by CQ treatment were greatly expanded in TAZ KO cells compared to the WT control (Fig. 6E). The average sizes of LC3B-positive autophagosomes and autolysosomes were significantly increased in TAZ deficiency, which was returned to normal by TAZ restoration, and even when autophagy vesicles were expanded by CQ treatment (Fig. 6F and G). These results indicate that TAZ deficiency promotes the autophagy pathway while accumulating altered autophagy vesicles.

### TAZ deficiency increases lysosomal biogenesis but impairs lysosomal function

**Figure 7:**
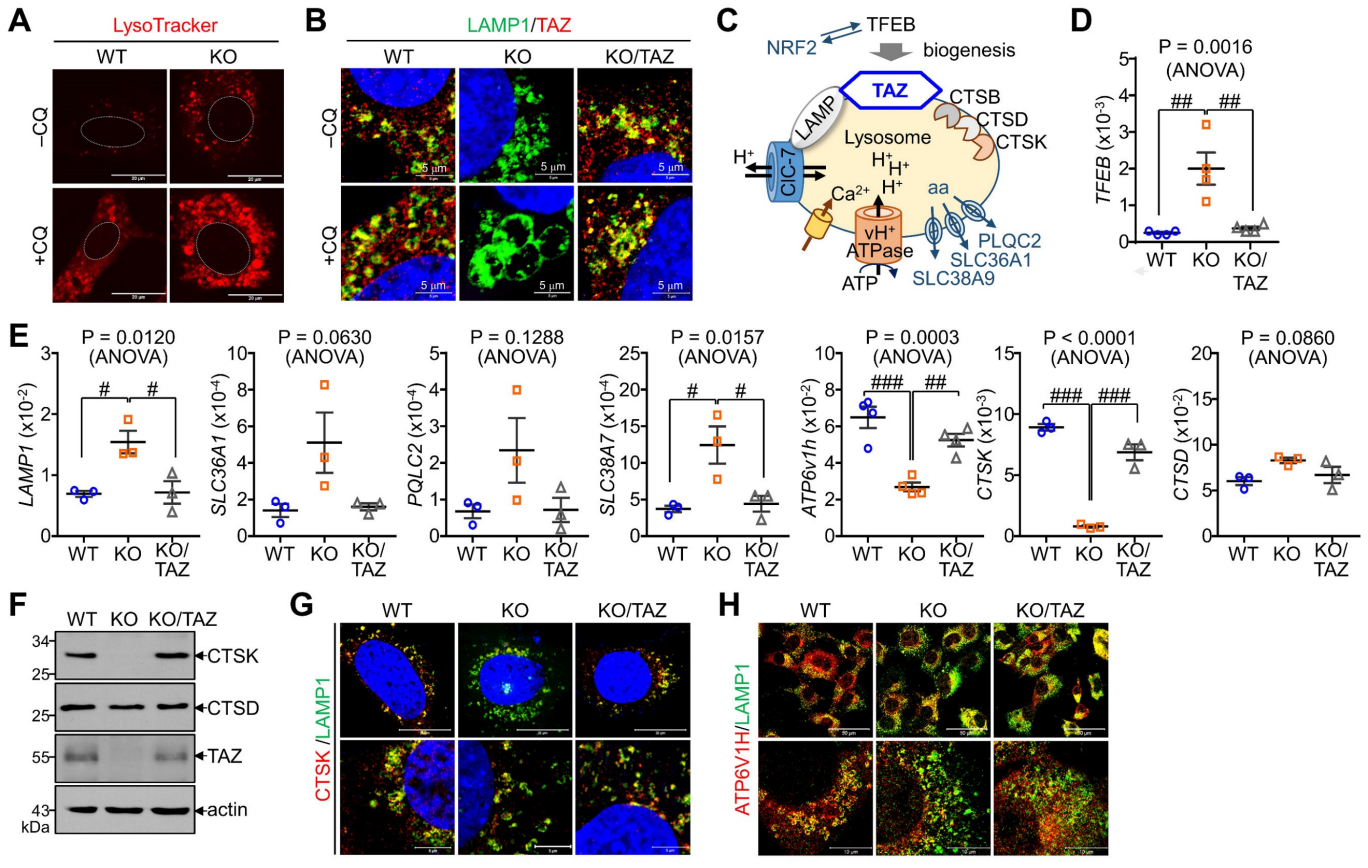

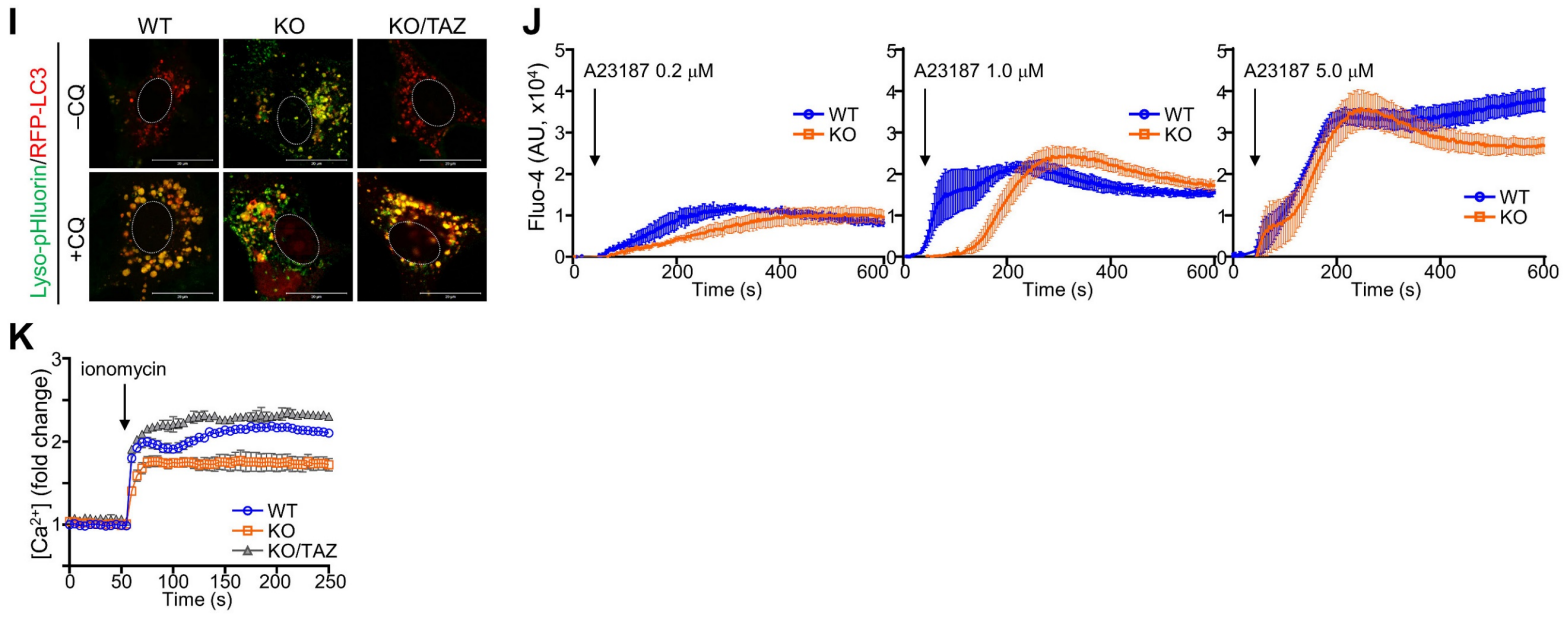
** Aberrant lysosomal expansion and dysfunction in TAZ deficiency.** (**A**) WT and KO MEF cells were treated with either vehicle or CQ (50 μM) for 4 h and subjected to LysoTracker staining. (**B**) WT, KO, and KO/TAZ MEF cells were incubated with or without CQ (50 μM) for 4 h and subjected to IF staining of LAMP1 and TAZ. (**C**) Schematic illustration of lysosomes. (**D-E**) Total RNA was prepared from MEF cells and analyzed to determine the relative transcript levels of TFEB (**D**) and lysosomal genes (**E**). (**F-H**) WT, KO, and KO/TAZ MEF cells were cultured under glucose-free conditions and subjected to IB analysis for CTSK, CTSD, TAZ, and actin (**F**) and IF staining for CTSK/LAMP1 (**G**) and ATP6V1H/LAMP (**H**). (**I**) WT, KO, and KO/TAZ MEF cells were transfected with lyso-pHluorin and RFP-LC3 vectors, followed by fluorescence confocal microscopy. (**J**) WT, KO, and KO/TAZ MEF cells were labeled with Fluo-4 AM (1 μM) and subjected to fluorometric analysis to analyze intracellular calcium concentration upon stimulation with A23187 (0.2, 1.0, and 5.0 μM, **J**) or ionomycin (0.5 μM, **K**). Representative images of at least three independent experiments are shown. Data are expressed as mean ± SEM. #P < 0.05; ##P< 0.005; ###P< 0.0001 by ANOVA with Tukey's HSD post-hoc test.

In addition to autophagy regulation, we sought to determine changes in NRF2-mediated lysosomal biogenesis. LysoTracker-labeled lysosomal vesicles were increased in TAZ KO cells, which was more pronounced by CQ treatment (Fig. 7A). Interestingly, TAZ was found to be expressed in lysosomes by co-localizing with LAMP1, and TAZ deficiency resulted in enlarged LAMP1-positive lysosomal vesicles, which were returned to similar levels in KO/TAZ as in WT cells. This abnormal enlargement of LAMP1-positive lysosomal vesicles was more evident in TAZ KO cells upon treatment with CQ (Fig. 7B). Consistent with enlarged lysosomes, expression of TFEB, a master regulator of lysosomal biogenesis that is increased by NRF2 37, was transcriptionally upregulated in TAZ KO cells, which was suppressed by TAZ restoration (Fig. 7C and D). Lysosomal genes, such as LAMP1 and amino acid transporters, were consequently increased in TAZ KO cells and normal in KO/TAZ cells (Fig. 7D), indicating a regulation of NRF2-TFEB-mediated lysosomal biogenesis by TAZ. Interestingly, we also found that the expression of proton transporter ATP6V1H and CTSK was significantly decreased in TAZ KO cells and returned to normal in KO/TAZ cells, suggesting an NRF2-independent TAZ function in lysosomes (Fig. 7E). Similar to transcript levels, CTSK protein level was drastically reduced in TAZ deficiency and rescued by forced TAZ expression in KO cells, whereas lysosomal cathepsin D was unaffected by TAZ depletion (Fig. 7F and G). In addition, the expression of ATP6V1H, a proton transporter important for lysosomal acidification, was reduced in TAZ KO cells, as were vesicles co-localized with LAMP1, and these changes were partially restored to normal in KO/TAZ cells (Fig. 7H). Further analysis of lysosomal acidification using the pH-sensitive Lyso-pHluorin, showing no fluorescence in the acidic lysosome, confirmed that the green fluorescence of the Lyso-pHluorin was prominent in the lysosomes of TAZ KO cells but not WT and KO/TAZ cells (Fig. 7I), indicating a lysosomal dysfunction in TAZ deficiency.

We also analyzed intracellular calcium concentration in response to calcium ionophore treatment. A marked increase in intracellular calcium concentration was observed in WT cells in response to A23187, an ionophore and lysosomal rupture inducer, whereas TAZ deficiency impaired intracellular calcium mobilization (Fig. 7J). Furthermore, the decrease in intracellular calcium mobilization was restored in KO/TAZ cells, which was found to be similar to WT cells (Fig. 7K). These results suggest that TAZ is indispensable for controlling lysosomal biogenesis and function.

### TAZ deficiency induces an abnormal autophagy-lysosomal pathway and leads to lung injury *in vivo*

**Figure 8:**
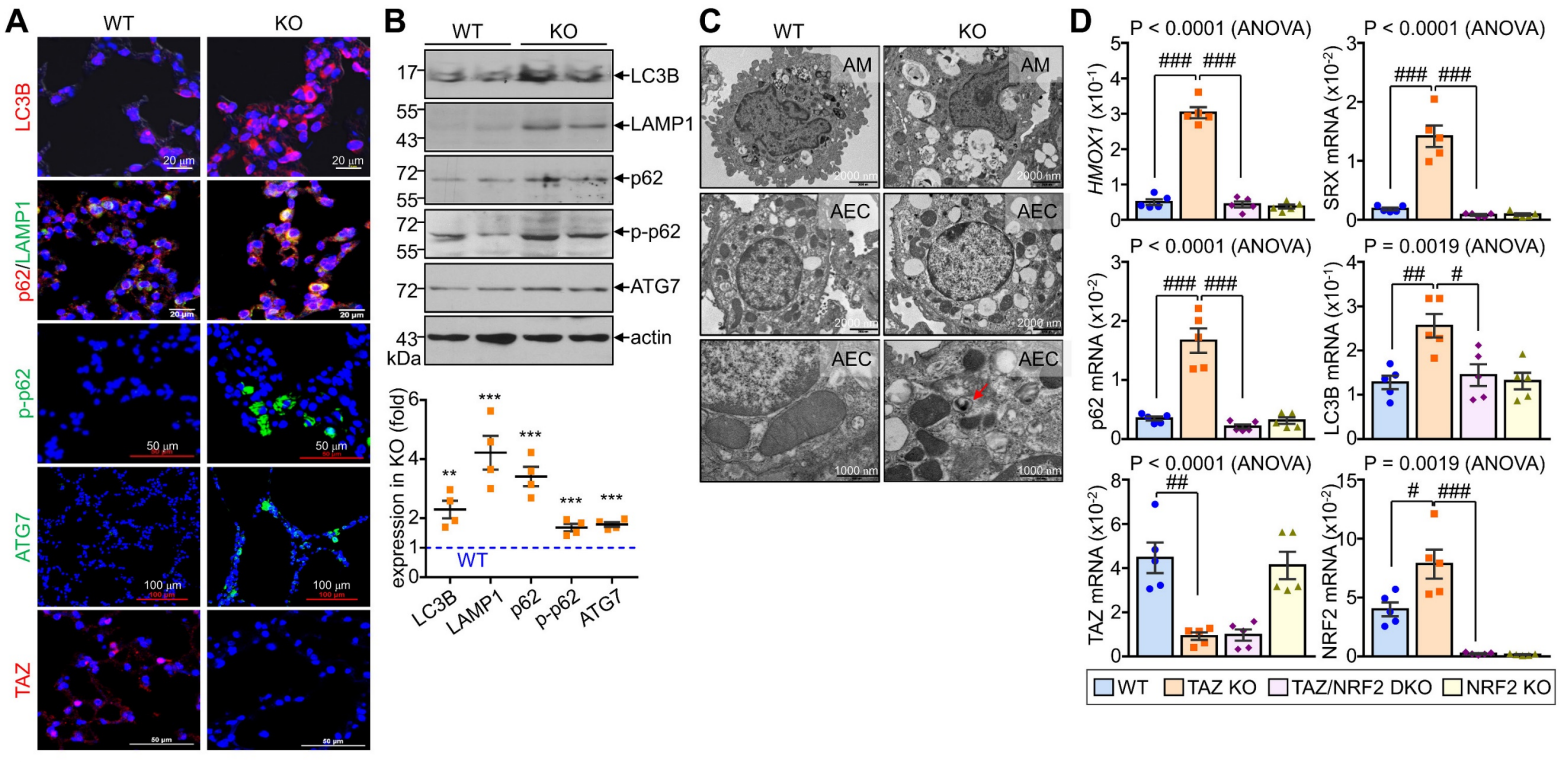

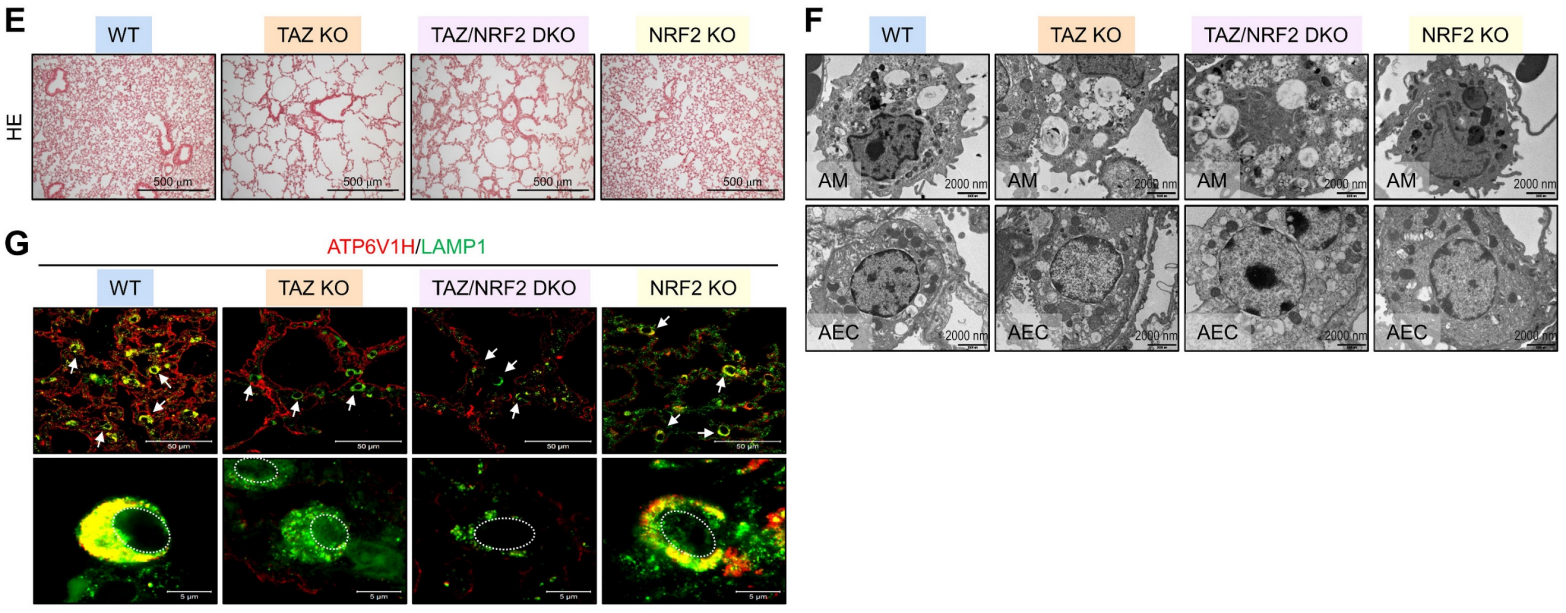
**Dysregulated autophagy-lysosomal pathway by TAZ deficiency.** Lung tissues were obtained from WT and TAZ KO mice (n = 4-5 per group). (**A**) Lung tissue sections were prepared and stained with antibodies against LC3B, p62, pp62, LAMP1, ATG7, and TAZ. (**B**) Protein lysates were extracted from the lung tissues and analyzed by IB and quantitative analysis. **P < 0.005; ***P < 0.0005 by two-tailed Student's t-test. (**C**) Electron microscopy images of alveolar macrophages (AM) and airway epithelial cells (AEC) in WT and KO lungs. (**D-G**) Lung tissues were harvested from WT, TAZ KO, TAZ/NRF2 DKO, and NRF2 KO mice and subjected to analysis of relative transcript levels of HMOX1, p62, TAZ, and NRF2 (**D**), HE staining (**E**), electron microscopy (**F**), and IF staining for ATP6V1H and LAMP1 (**G**). Representative images of at least three independent experiments are shown. Data are expressed as mean ± SEM. #P < 0.05; ##P< 0.005; ###P< 0.0001 by ANOVA with Tukey's HSD post-hoc test.

We investigated the *in vivo* effects of TAZ deficiency on the autophagy-lysosomal pathway by analyzing the lungs of WT and TAZ KO mice. As expected, autophagy-lysosomal markers, including LC3B, p62, phosphorylated p62, LAMP1, and ATG7, were significantly increased in TAZ KO lungs (Fig. 8A). Consistently, protein levels of autophagy and lysosomal markers were quantitatively increased in TAZ KO lungs (Fig. 8B). Electron microscopy revealed an increased number of vacuolated cells in the lungs of TAZ KO mice. Autophagy vesicles, lysosomes, and lamellar inclusion bodies were more frequently observed in alveolar macrophages and airway epithelial cells of TAZ KO mice compared to WT control (Fig. 8C). To investigate whether these abnormalities in TAZ deficiency could be rescued by NRF2 ablation, we generated TAZ and NRF2 double deficient (TAZ/NRF2 DKO) mice. The relative transcripts of NRF2 targets, which were significantly elevated in TAZ-deficient lungs, were drastically reduced by further NRF2 depletion (Fig. 8D), but the lung injury observed in TAZ KO mice was still seen in the lungs of TAZ/NRF2 DKO mice (Fig. 8E). In addition, the abnormal autophagy vesicles in TAZ KO were more clearly observed in TAZ/NRF2 DKO mice lungs and were characterized by a sharp decrease in ATP6V1H and LAMP1 (Fig. 8F and G). These results suggest that both TAZ and NRF2 are essential in the autophagy-lysosomal pathway, and failure of their complementary regulation can cause severe cell and tissue damage.

### TAZ deficiency increases proteotoxic and oxidative stress *in vitro* and *in vivo*

**Figure 9:**
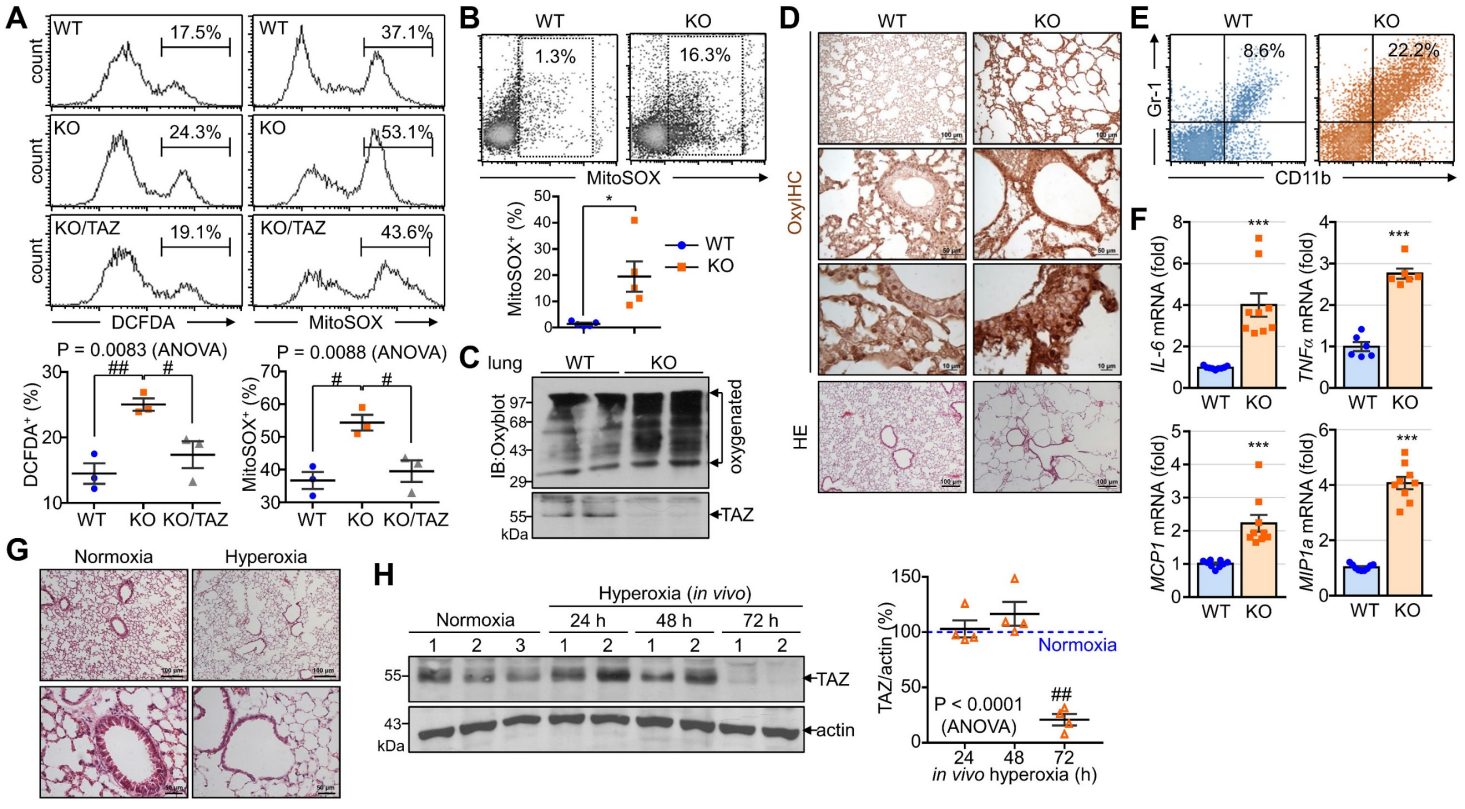

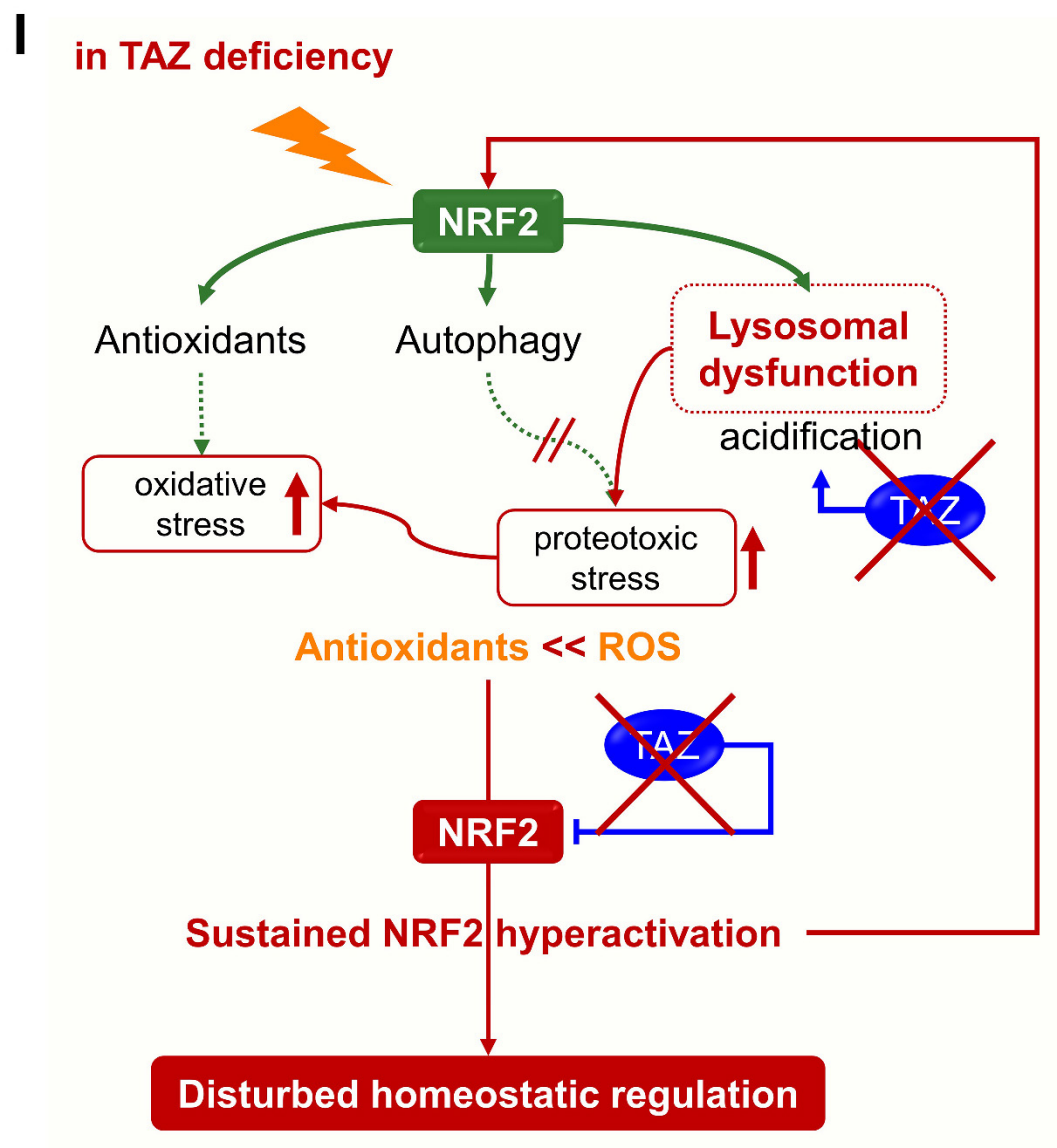
** Accumulation of proteotoxic and oxidative stress due to TAZ deficiency.** (**A**) WT, KO, and KO/TAZ MEF cells were incubated in glucose-free conditions before harvest and stained with either cell-permeable DCFDA or MitoSOX. DCFDA- or MitoSOX-positive cells were quantitatively analyzed by flow cytometry and CellQuest software. #P < 0.05; ##P< 0.005 by ANOVA with Tukey's HSD post-hoc test. (**B**-**F**) Lung tissues were harvested from WT and TAZ KO mice (n = 5-9 per group). Single-cell suspensions were freshly isolated from WT and TAZ KO mice lungs and immediately stained with MitoSOX, followed by flow cytometry analysis (**B**). *P < 0.05 by two-tailed Student's t-test. Protein extracts were harvested from WT and TAZ KO mice lungs and immediately subjected to IB analysis of oxygenated proteins using Oxyblot (**C**). Lung tissue sections were freshly prepared and subjected to OxyIHC staining (**D**). Gr-1^+^CD11b^+^ myeloid cells were quantitatively analyzed using single-cell suspensions of lung tissues by flow cytometry (**E**). The relative transcript level of inflammation mediators in WT and KO mice lungs (**F**). ***P < 0.0005 by two-tailed Student's t-test. (**G-H**) Mice were exposed to normal oxygen concentration (normoxia) or 95% oxygen conditions (hyperoxia) for 24 h, 48 h, and 72 h, and lung tissues were analyzed by HE staining (**G**) and IB and quantitative analysis for TAZ (**H**). (**I**) A working model for the accumulation of proteotoxic and oxidative stress due to TAZ deficiency. Representative images from at least three independent experiments are presented. ##P< 0.005 by ANOVA with Tukey's HSD post-hoc test.

The accumulation of abnormal autophagy vesicles in TAZ deficiency led us to measure intracellular oxidative and proteotoxic stress *in vitro* and *in vivo*. Intracellular ROS levels increased in WT MEF cells under glucose-deprived conditions, were more significantly increased in TAZ KO cells, and decreased to control levels in KO/TAZ cells (Fig. 9A). Consistent with the *in vitro* results, intracellular ROS levels were higher in the lungs of TAZ KO mice than in WT lungs (Fig. 9B). In addition, Oxyblot and OxyIHC analyses showed that ROS-induced carbonyl oxidation of proteins was further increased in TAZ KO lungs, accompanied by lung injury such as enlarged air spaces and destruction of lung epithelial cells (Fig. 9C and D). Consequently, injury in TAZ KO lungs was accompanied by CD11b-positive macrophage infiltration and increased production of inflammatory cytokines (Fig. 9E and F). Indeed, when hyperoxia caused severe lung injury and rapid death, TAZ expression decreased dramatically, suggesting an important role for TAZ in overcoming external stress (Fig. 9G and H). These results suggest that TAZ deficiency fails to modulate NRF2 activity and maintain lysosomal function, dysregulating the autophagy-lysosomal pathway, accumulating proteotoxic and oxidative stress, and ultimately leading to cell and tissue damage (Fig. 9I).

## Discussion

Our results showed that TAZ directly interacts with NRF2 to control NRF2 hyperactivation through inhibition of the DNA-binding and transcriptional activities and plays crucial roles in the regulation of cellular homeostasis by modulating autophagy-lysosomal pathway in both NRF2-dependent and -independent ways.

TAZ deficiency sustained the activation of NRF2 and upregulated the NRF2-mediated antioxidant and autophagy-lysosomal pathways. Although TAZ deficiency increased the expression of antioxidants, intracellular ROS was rather elevated in TAZ deficiency *in vitro* and *in vivo*, possibly resulting in or due to abnormal expansion and accumulation of autophagy vesicles. It is well known that oxidative stress activates autophagy, a major cellular defense against oxidative stress, and that altered autophagy pathways are associated with the cellular oxidative stress and pathological process of lung diseases (Oranatowsk et al., 2020; Moreno et al., 2018). The altered autophagy process in TAZ deficiency was consistent with the previous finding that double deficiency of TAZ and YAP increased autophagosome accumulation 53. In addition, it has been reported that contact inhibition at high cell density induced autophagy by suppressing TAZ/YAP expression in cancer cells, and TAZ/YAP knockdown inhibited autophagosome formation in low-density cancer cells 54. The commonality of these controversial findings, including our results, suggests that TAZ is indispensable for the normal process of the autophagy-lysosomal degradation pathway and cell homeostatic control.

TAZ was essential not only for optimal regulation of NRF2 activity but also for NRF2-dependent and -independent induction of lysosomal expansion, acidification, and function. TAZ was expressed in lysosomes by colocalizing with LAMP1, and its deficiency increased the number and size of lysosomal vesicles. Recently, it was reported that failure of NRF2 regulation by KEAP1 deficiency caused aberrant lysosomal biogenesis through activation of TFEB, resulting in aberrant lysosomal accumulation and liver abnormalities during embryogenesis 37. TAZ deficiency also increased the expression of TFEB and promoted the expression of lysosomal amino acid transporters due to persistent NRF2 activation. These results strongly support the idea that TAZ acts as an inhibitor of NRF2 during the homeostatic control of lysosomal biogenesis. Apart from lysosomal biogenesis, TAZ deficiency impaired lysosomal acidification and lysosomal calcium release, leading to lysosomal dysfunction. Since lysosomal pH is primarily maintained and controlled by the activity of vacuolar ATPase 55, the decrease in expression of vacuolar ATPase (ATP6V1H) in TAZ deficiency likely caused defective lysosomal pH and lysosomal enzyme functions. Additional NRF2 depletion in TAZ deficiency normalized the increased expression of HMOX1 and p62 to basal levels, but TAZ and NRF2 double deficiency further expanded and accumulated aberrant vesicles in the lung, suggesting the possibility of NRF2-dependent and NRF2-independent regulation of the autophagy-lysosomal pathway. In other words, it is proposed that TAZ is involved in the regulation of lysosomal biogenesis in an NRF2-dependent manner and regulates lysosomal activity by regulating the expression of ATP6V1H and CTSK in an NRF2-independent manner. However, the exact molecular mechanisms underlying how TAZ regulates the expression of ATP6V1H and CTSK remain to be elucidated in the future. Based on the importance of TAZ in regulating the autophagy-lysosomal pathway, it will be interesting to investigate the correlation between TAZ expression and human diseases associated with abnormal autophagy-lysosomal pathways, such as lysosomal storage diseases and to verify whether TAZ has a beneficial effect in improving lysosomal function and treating lysosomal storage diseases.

## Materials and Methods

### Materials

SFN and MG132 were purchased from Sigma-Aldrich (St. Louis, MO, USA). All antibodies were purchased from Abcam (Cambridge, MA, USA), Abfrontier (Seoul, Korea), BD Biosciences (San Jose, CA, USA), Cell Signaling Technology (Danvers, MA, USA), MBL International (Woburn, MA, USA), Merck Millipore (Burlington, MA, USA), Novus Biologicals (Littleton, CO, USA), and Santa Cruz Biotechnology Inc. (Santa Cruz, CA, USA). Antibodies for IB, IF, IP, and IHC are listed in Table S1 in the Supplementary Information. Pre-stained protein standards (180, 130, 95, 72, 55, 43, 34, 25, and 17 kDa, Bio-Rad Laboratories, Hercules, CA, USA) were used for size estimation.

### Animals and hyperoxia-induced lung injury model

Male WT C57BL/6 mice were purchased from the Jackson Laboratory (Minneapolis, MN, USA), and TAZ KO mice were generated as previously reported 7. NRF2 KO mice were obtained from RIKEN BioResource Center (Tsukuba, Japan), and TAZ/NRF2 DKO mice were generated by crossing TAZ KO and NRF2 KO mice. All mice were housed under controlled conditions in a specific pathogen-free animal facility at Ewha Womans University. All animal care and experiments were approved by IACUC (2010-13-3, 2015-01-020, and 16-026) at Ewha Womans University and conducted according to the IACUC guidelines. Male WT mice were divided into two groups and exposed to either normal oxygen conditions (normoxia) or hyperoxic conditions of 95% oxygen that induce acute lung injury and increase lethality 56.

### Protein-protein interaction

Highly transfectable human embryonic kidney (HEK)-293T cells (American Type Culture Collection, Manassas, VA, USA) were transfected with different combinations of expression vectors such as Flag-tagged TAZ truncations and Myc- or GFP-tagged NRF2 truncations using the calcium phosphate transfection method 57. Cell lysates were harvested and immunoprecipitated with Flag-M2 agarose beads overnight, followed by SDS-PAGE and IB analysis using antibodies against Myc (9E10) or GFP (B-2). For endogenous protein interaction assay, mouse lung extracts of WT and TAZ KO mice and protein lysates of WT and KO MEF cells were incubated with anti-TAZ antibody and subsequently protein A/G-agarose bead. Immune complexes were subjected to IB analysis using antibodies against NRF2 and TAZ.

### Reporter assay

HEK-293T cells were transiently transfected with the reporter gene (p*SRX*-luc, p*GSTA*-luc, or p*HMOX1*-luc) and expression vectors (NRF2 or TAZ truncations). The pCMVβ vector was also transfected for normalization of the transfection efficiency. Cell lysates were assayed with a luciferase assay kit (Promega, Madison, WI, USA) and a β-galactosidase assay kit (Thermo Fisher Scientific, Waltham, MA, USA) using a luminometer equipped at Ewha Drug Development Research Core Center. Relative activity was expressed as the fold change compared to the control. All reporter assays were repeated at least three times for statistical evaluation.

### DNA pulldown assay

HEK-293T cells were transfected with different concentrations of the Flag-tagged TAZ expression vector and NRF2 expression vector. Cell lysates were harvested in HKMG buffer and incubated with biotinylated double-stranded DNA (SRX-ARE wt, SRX-ARE mt, or NQO1-ARE), followed by precipitation using streptavidin-agarose beads. Protein-DNA complexes were resolved by SDS-PAGE and analyzed by IB analysis using anti-NRF2 antibody. Oligonucleotides for *SRX*-ARE wt, *SRX*-ARE mt, and *NQO1*-ARE were synthesized as provided in Table S2.

### ChIP and quantitative PCR

The ChIP assay was conducted in mouse lung tissues and MEF cells of WT and TAZ KO mice. Lung tissues were harvested from WT and TAZ KO mice and fixed in formaldehyde. MEF cells were cultured and treated with SFN (10 µM), followed by 1% formaldehyde for 10 min for the ChIP assay. The ChIP assay was performed using the Magna ChIP™ A/G Chromatin Immunoprecipitation kit according to the manufacturer′s instructions (Merck Millipore). Nuclear lysates were prepared and sonicated on ice to shear DNA, followed by incubation with NRF2 antibody (ab62352) and protein A/G magnetic beads (Thermo Fisher Scientific). Chromatin DNA was eluted in the ChIP elution buffer containing proteinase K and subjected to semi-quantitative PCR at different cycle numbers 25, 27, and 29 or quantitative PCR analysis using SyBr Green-based real-time PCR. The ChIP-qPCR primers for *SRX* and *NQO1* genes are listed in Table S2.

### Reverse transcription and real-time polymerase chain reaction (PCR)

Total RNA was isolated from the lung tissues and MEF cells using TRIzol reagent and subjected to reverse transcription using a cDNA synthesis kit (Thermo Fisher Scientific). Quantitative real-time PCR analysis was performed using the StepOnePlus system (Applied Biosystems, Foster City, CA, USA). The relative expression level was calculated after normalization to the level of β-actin. The primer sequences used are presented in Table S2.

### Fluorescence staining and HCS analysis

For IF staining, MEF cells were cultured on pre-coated cover glasses and subjected to fixation. The glass slides were incubated overnight in the dark with fluorescence-tagged antibodies against NRF2 and TAZ and counterstained with DAPI for 5 min. For LysoTracker staining, the slides were stained with LysoTracker Red DND-99 (50 nM; Thermo Fisher Scientific). The slides were then mounted with a fluorescence mounting medium (DAKO Omnis; Agilent Technologies Inc., Santa Clara, CA, USA) and observed under a confocal laser microscope (Nikon A1R). For the HCS assay, cells were plated onto the HCS-based plates and stained with slides were fluorescence-tagged antibodies against LC3B, followed by HCS platform analysis. Automated cellular imaging and fluorescence intensity of cellular puncta were quantitatively analyzed using the CellInsight CX5 HCS Platform (Thermo Fisher Scientific).

### Hematoxylin and eosin (HE) staining and immunohistochemistry (IHC)

Lung tissues were isolated from WT and TAZ KO mice (2-4 months old, male) and fixed in 10% neutralized formalin. Tissues were sectioned at a thickness of 4 µm and stained with HE (Sigma-Aldrich). Stained sections were analyzed using a Nikon Eclipse E200 microscope (Nikon, Tokyo, Japan) with an attached digital camera (Olympus, Melville, NY, USA). For IHC analysis, tissue sections were sequentially incubated with the specific primary and fluorescence-conjugated secondary antibodies (Molecular Probes, Eugene, OR, USA). Slides were counterstained with 4, 6-diamidino-2-phenylindole (DAPI) for nuclear staining and observed under a confocal microscope (Nikon A1R; Nikon) and NIS-Elements AR software.

### Measurement of autophagosomes and autolysosomes

According to the methods for monitoring autophagic flux 58, WT, TAZ KO, and KO/TAZ MEF cells were seeded in a 24-well plate on a cover slide and transfected with the mRFP-GFP-LC3 vector for 24 h. Cells were cultured in the presence or absence of CQ (50 μM) for 4 h and then washed, followed by fixation in 4% paraformaldehyde. Fluorescence images were acquired under a confocal fluorescence microscope (ZEISS LSM 880 with Airyscan, Carl Zeiss AG, Oberkochen, Germany) at Ewha Fluorescence Imaging Core Center. The red and green fluorescence intensity and RFP^+^GFP^+^ (yellow) and RFP^+^ (red) puncta were determined in triplicates by counting a total of 25 areas through automated HCS platforms (CellInsight CX7 LZR High-Content Screening Platform, Thermo Fisher Scientific).

### Intracellular calcium assay

WT, TAZ KO, and KO/TAZ MEF cells were cultured in DMEM supplemented with 10% FBS and were incubated with Fluo-4 AM (1 μM, Thermo Fisher Scientific) for 30 min. Cells were washed and immediately subjected to fluorescence monitoring (Molecular Devices, Sunnyvale, CA, USA). Fluorescence was monitored to ensure stable baselines before and after stimulation with ionomycin (0.5 μM, Thermo Fisher Scientific). The fluorescence was normalized to the baseline fluorescence measured at the first time point. The negative control wells had no treatment. Intracellular calcium change was expressed as fold induction compared to the fluorescence levels of untreated WT cells.

### Measurement of cellular ROS *in vivo* and *in vitro*

For measurement of the oxidative stress *in vivo*, lung tissues were isolated from WT and TAZ KO mice and treated with fresh methacarn fixative, followed by protein oxidation analysis using an OxyIHC Oxidative Stress Detection kit according to the manufacturer′s instructions (Merck Millipore, Darmstadt, Germany). Images were obtained using a microscope (Nikon Eclipse E200). For measuring mitochondrial ROS production, single-cell suspensions of lung tissues were prepared 59 and incubated with MitoSOX Red reagent (5 μM, Thermo Fisher Scientific, San Diego, CA, USA) for 20 min at 37°C in the dark, followed by flow cytometry analysis and quantitation (BD Biosciences). For protein oxidation analysis, protein extracts were harvested from the lungs and analyzed by sodium dodesyl sulfate (SDS)-polyacrylamide gel electrophoresis (PAGE) and with the OxyBlot protein oxidation kit (Merck Millipore). For measurement of cellular ROS *in vitro*, WT, TAZ KO, and KO/TAZ MEF cells were cultured in complete DMEM and harvested in phosphate-buffered saline. The cells were then incubated with chloromethyl-dichlorodihydrofluorescein diacetate (CM-DCFDA, 10 μM) and MitoSOX Red reagent (5 μM, Thermo Fisher Scientific) for measuring cellular and mitochondrial ROS, respectively. Cells were stained for 10 min and subjected to flow cytometry and CellQuest software analysis (BD Biosciences).

### Electron microscopy

WT and TAZ KO mouse lungs (n = 3 per group) were isolated and fixed in 2.5% glutaraldehyde at pH 7.2 for 3 h and postfixed in 1% OsO4 in 0.1 M cacodylate buffer for 1 h. Lung tissue was dehydrated in graded alcohols and embedded in epoxy resin, and tissue sections were cut at 60 nm with a diamond knife, stained with 5% uranyl acetate for 10 min and 1% lead citrate for 5 min, and subjected to transmission electron microscopic observation. All digital images were obtained using a JEM 1016CX electron microscope with a digital camera (JEOL Ltd, Tokyo, Japan).

### Statistical analysis

All experiments were performed at least three times, and data are expressed as mean ± standard error of the mean (SEM). All statistical analysis was performed using Prism 7.0 (GraphPad Software Inc., San Diego, CA, USA). The statistical significance of mean differences was analyzed using two-tailed Student's *t*-test or one-way analysis of variance (ANOVA). P values less than 0.05 were considered to be statistically significant.

## Supplementary Material

Supplementary tables.

## Abbreviations

ARE: antioxidant-responsive element
ATG: autophagy-related gene
ATP6V1H: ATPase H+ Transporting V1 Subunit H
CAT: catalase
CD11b: Cluster of differentiation 11 b
CTSD: cathepsin D
CTSK: cathepsin K
DCFDA: dichlorodihydrofluorescein
GSTA: glutathione S-transferase A
HE: hematoxylin & eosin
IL-6: interleukin-6
HMOX1: heme oxygenase-1
KO: knockout
LAMP1: lysosomal associated membrane protein 1
LC3B: Microtubule-associated protein 1 light chain 3 B
MCP1: monocyte chemotaxis protein 1
MIP1a: macrophage inflammatory protein 1 alpha
MEF: mouse embryonic fibroblast
NRF2: nuclear factor erythroid 2-related factor 2
NQO1: NAD(P)H quinone oxidoreductase 1
PQLC: PQ loop repeat-containing transporter
ROS: reactive oxygen species
SFN: sulforaphane
SLC: solute carrier transporter
SRX: sulfiredoxin
TAZ: transcriptional coactivator with PDZ-binding motif
TFEB: transcription factor EB
TNF: tumor necrosis factor
WT: wild-type

## References

[1] Yu FX, Zhao B, Guan KL (2015). Hippo Pathway in Organ Size Control, Tissue Homeostasis, and Cancer. Cell.

[2] Hansen CG, Moroishi T, Guan KL (2015). YAP and TAZ: a nexus for Hippo signaling and beyond. Trends Cell Biol.

[3] Park KS, Whitsett JA, Di Palma T (2004). TAZ interacts with TTF-1 and regulates expression of surfactant protein-C. J Biol Chem.

[4] Hong JH, Hwang ES, McManus MT (2005). TAZ, a transcriptional modulator of mesenchymal stem cell differentiation. Science.

[5] Mitani A, Nagase T, Fukuchi K (2009). Transcriptional coactivator with PDZ-binding motif is essential for normal alveolarization in mice. Am J Respir Crit Care Med.

[6] Park GH, Jeong H, Jeong MG (2014). Novel TAZ modulators enhance myogenic differentiation and muscle regeneration. Br J Pharmacol.

[7] Jeong MG, Song H, Shin JH (2017). Transcriptional coactivator with PDZ-binding motif is required to sustain testicular function on aging. Aging Cell.

[8] Shin JH, Lee G, Jeong MG (2020). Transcriptional coactivator with PDZ-binding motif suppresses the expression of steroidogenic enzymes by nuclear receptor 4 A1 in Leydig cells. FASEB J.

[9] Li Y, Yang S, Qin L (2021). TAZ is required for chondrogenesis and skeletal development. Cell Discov.

[10] Makita R, Uchijima Y, Nishiyama K (2008). Multiple renal cysts, urinary concentration defects, and pulmonary emphysematous changes in mice lacking TAZ. Am J Physiol Renal Physiol.

[11] Konishi T, Schuster RM, Lentsch AB (2018). Proliferation of hepatic stellate cells, mediated by YAP and TAZ, contributes to liver repair and regeneration after liver ischemia/reperfusion injury. Am J Physiol Gastrointest Liver Physiol.

[12] Yui S, Azzolin L, Maimets M (2018). YAP/TAZ-Dependent Reprogramming of Colonic Epithelium Links ECM Remodeling to Tissue Regeneration. Cell stem cell.

[13] Kim AR, Park JI, Oh HT (2019). TAZ stimulates liver regeneration through interleukin-6-induced hepatocyte proliferation and inhibition of cell death after liver injury. FASEB J.

[14] LaCanna R, Liccardo D, Zhang P (2019). Yap/Taz regulate alveolar regeneration and resolution of lung inflammation. J Clin Invest.

[15] Sun T, Huang Z, Zhang H (2019). TAZ is required for lung alveolar epithelial cell differentiation after injury. JCI Insight.

[16] Hicks-Berthet J, Ning B, Federico A (2021). Yap/Taz inhibit goblet cell fate to maintain lung epithelial homeostasis. Cell Rep.

[17] Hwang JH, Heo W, Park JI (2023). Endothelial TAZ inhibits capillarization of liver sinusoidal endothelium and damage-induced liver fibrosis via nitric oxide production. Theranostics.

[18] Hwang JH, Kim AR, Kim KM (2019). TAZ couples Hippo/Wnt signalling and insulin sensitivity through Irs1 expression. Nat Commun.

[19] Hwang JH, Kim KM, Oh HT (2022). TAZ links exercise to mitochondrial biogenesis via mitochondrial transcription factor A. Nat Commun.

[20] Kim KM, Yoo GD, Heo W (2023). TAZ stimulates exercise-induced muscle satellite cell activation via Pard3-p38 MAPK-TAZ signalling axis. J Cachexia Sarcopenia Muscle.

[21] Jeong M, Kim H, Hwang E (2021). The essential role of TAZ in normal tissue homeostasis. Archives of Pharmacal Research.

[22] Zanconato F, Cordenonsi M, Piccolo S (2019). YAP and TAZ: a signalling hub of the tumour microenvironment. Nat Rev Cancer.

[23] Chan SW, Lim CJ, Guo K (2008). A role for TAZ in migration, invasion, and tumorigenesis of breast cancer cells. Cancer Res.

[24] Bartucci M, Dattilo R, Moriconi C (2015). TAZ is required for metastatic activity and chemoresistance of breast cancer stem cells. Oncogene.

[25] Janse van Rensburg HJ, Azad T, Ling M (2018). The Hippo pathway component TAZ promotes immune evasion in human cancer through PD-L1. Cancer Res.

[26] Noguchi S, Saito A, Mikami Y (2017). TAZ contributes to pulmonary fibrosis by activating profibrotic functions of lung fibroblasts. Sci Rep.

[27] Noguchi S, Saito A, Horie M (2014). An integrative analysis of the tumorigenic role of TAZ in human non-small cell lung cancer. Clin Cancer Res.

[28] Lignitto L, LeBoeuf SE, Homer H (2019). Nrf2 Activation Promotes Lung Cancer Metastasis by Inhibiting the Degradation of Bach1. Cell.

[29] Singh A, Daemen A, Nickles D (2021). NRF2 Activation Promotes Aggressive Lung Cancer and Associates with Poor Clinical Outcomes. Clin Cancer Res.

[30] Dodson M, Redmann M, Rajasekaran NS (2015). KEAP1-NRF2 signalling and autophagy in protection against oxidative and reductive proteotoxicity. Biochem J.

[31] Zhang DD, Hannink M (2003). Distinct cysteine residues in Keap1 are required for Keap1-dependent ubiquitination of Nrf2 and for stabilization of Nrf2 by chemopreventive agents and oxidative stress. Mol Cell Biol.

[32] Furukawa M, Xiong Y (2005). BTB protein Keap1 targets antioxidant transcription factor Nrf2 for ubiquitination by the Cullin 3-Roc1 ligase. Mol Cell Biol.

[33] Saito R, Suzuki T, Hiramoto K (2016). Characterizations of Three Major Cysteine Sensors of Keap1 in Stress Response. Mol Cell Biol.

[34] Suzuki T, Muramatsu A, Saito R (2019). Molecular Mechanism of Cellular Oxidative Stress Sensing by Keap1. Cell Rep.

[35] Jain A, Lamark T, Sjottem E (2010). p62/SQSTM1 is a target gene for transcription factor NRF2 and creates a positive feedback loop by inducing antioxidant response element-driven gene transcription. J Biol Chem.

[36] Tonelli C, Chio IIC, Tuveson DA (2018). Transcriptional Regulation by Nrf2. Antioxid Redox Signal.

[37] Ong AJS, Bladen CE, Tigani TA (2023). The KEAP1-NRF2 pathway regulates TFEB/TFE3-dependent lysosomal biogenesis. Proc Natl Acad Sci U S A.

[38] Komatsu M, Kurokawa H, Waguri S (2010). The selective autophagy substrate p62 activates the stress responsive transcription factor Nrf2 through inactivation of Keap1. Nat Cell Biol.

[39] Inami Y, Waguri S, Sakamoto A (2011). Persistent activation of Nrf2 through p62 in hepatocellular carcinoma cells. J Cell Biol.

[40] Inoue D, Suzuki T, Mitsuishi Y (2012). Accumulation of p62/SQSTM1 is associated with poor prognosis in patients with lung adenocarcinoma. Cancer Sci.

[41] Jiang T, Harder B, Rojo de la Vega M (2015). p62 links autophagy and Nrf2 signaling. Free Radic Biol Med.

[42] Racanelli AC, Kikkers SA, Choi AMK (2018). Autophagy and inflammation in chronic respiratory disease. Autophagy.

[43] Klionsky DJ, Petroni G, Amaravadi RK (2021). Autophagy in major human diseases. EMBO J.

[44] Fan Y, Shao J, Wei S (2021). Self-eating and Heart: The Emerging Roles of Autophagy in Calcific Aortic Valve Disease. Aging Dis.

[45] Lian YE, Bai YN, Lai JL (2022). Aberrant regulation of autophagy disturbs fibrotic liver regeneration after partial hepatectomy. Front Cell Dev Biol.

[46] Ichimura Y, Waguri S, Sou YS (2013). Phosphorylation of p62 activates the Keap1-Nrf2 pathway during selective autophagy. Mol Cell.

[47] DeNicola GM, Karreth FA, Humpton TJ (2011). Oncogene-induced Nrf2 transcription promotes ROS detoxification and tumorigenesis. Nature.

[48] Wang H, Liu X, Long M (2016). NRF2 activation by antioxidant antidiabetic agents accelerates tumor metastasis. Sci Transl Med.

[49] Ni HM, Woolbright BL, Williams J (2014). Nrf2 promotes the development of fibrosis and tumorigenesis in mice with defective hepatic autophagy. J Hepatol.

[50] Escoll M, Lastra D, Pajares M (2020). Transcription factor NRF2 uses the Hippo pathway effector TAZ to induce tumorigenesis in glioblastomas. Redox Biol.

[51] Yu HF, Zheng LW, Yang ZQ (2021). TAZ as a novel regulator of oxidative damage in decidualization via Nrf2/ARE/Foxo1 pathway. Exp Mol Med.

[52] Huang JC, Yue ZP, Yu HF (2022). TAZ ameliorates the microglia-mediated inflammatory response via the Nrf2-ROS-NF-kappaB pathway. Mol Ther Nucleic Acids.

[53] Totaro A, Zhuang Q, Panciera T (2019). Cell phenotypic plasticity requires autophagic flux driven by YAP/TAZ mechanotransduction. Proc Natl Acad Sci U S A.

[54] Pavel M, Renna M, Park SJ (2018). Contact inhibition controls cell survival and proliferation via YAP/TAZ-autophagy axis. Nat Commun.

[55] Mindell JA (2012). Lysosomal acidification mechanisms. Annu Rev Physiol.

[56] Tateda K, Deng JC, Moore TA (2003). Hyperoxia mediates acute lung injury and increased lethality in murine Legionella pneumonia: the role of apoptosis. J Immunol.

[57] Kim AR, Kim KM, Byun MR (2017). (-)-Epigallocatechin-3-gallate stimulates myogenic differentiation through TAZ activation. Biochem Biophys Res Commun.

[58] Mizushima N, Yoshimori T, Levine B (2010). Methods in mammalian autophagy research. Cell.

[59] Bantikassegn A, Song X, Politi K (2015). Isolation of epithelial, endothelial, and immune cells from lungs of transgenic mice with oncogene-induced lung adenocarcinomas. Am J Respir Cell Mol Biol.

