# Supplementary material for: TAZ deficiency impairs the autophagy-lysosomal pathway through NRF2 dysregulation and lysosomal dysfunction
Source: Int J Biol Sci. 2024 Apr 22;20(7):2592–606. doi: 10.7150/ijbs.88897 (PMC11077375; doi:10.7150/ijbs.88897)
Supplement: Supplementary file 1 — Supplementary tables. [file ijbsv20p2592s1.pdf]

TAZ deficiency impairs the autophagy-lysosomal pathway through NRF2 dysregulation and lysosomal dysfunction

Hyo Kyeong Kim[1], Hana Jeong[1], Mi Gyeong Jeong[1], Hee Yeon Won[1], Gibbeum Lee[1], Soo Han Bae[2], Miso Nam[3], Sung Hoon Lee[4], Geum-Sook Hwang[3], and Eun Sook Hwang[1,*]

Supplementary information

This file includes Tables S1−S2

Supplemental Table S1. Antibodies

Name	Supplier	Cat no.	Clone no.	Purpose
AMPK	Cell Signaling Technology	2532		IB
ATG7	Abcam	ab53325		IB & IHC
β-actin	Santa Cruz Biotechnology	sc-47778	C4	IB
CAT	Ab frontier	LF-MA0010	11A1	IB
CD11b	BD Pharmingen	561690	M1/70	FC
Flag	Sigma-Aldrich	F7425		IB
Flag-M2	Sigma-Aldrich	A2220		IP
GFP	Santa Cruz Biotechnology	sc-9696	B-2	IB
Gr-1	BD Pharmingen	553123	RB6-8C5	FC
HA	Santa Cruz Biotechnology	sc-7392	F-7	IB
HO-1	Abcam	ab13243		IB & IHC
Lamin	Abcam	ab 16048		IB
LAMP1	Santa Cruz Biotechnology	sc-19992	1D4B	IB & IHC
LC3	Sigma-Aldrich	L8919		IB
LC3B	Cell Signaling	3868P	D11	IHC
Myc	Santa Cruz Biotechnology	sc-40	9E10	IB
NRF2	Abcam	ab62352<br>ab31163	EP1808Y	IP & ChIP<br>IHC
OxyBlot	MERCK Millipore	S7150		IB
OxyIHC	MERCK Millipore	S7450		IHC
p62	MBL	PM045		IB & IHC
p-p62 (S-352)	MBL	PM074		IB & IHC
p-AMPK	Cell Signaling Technology	2531		IB
SRX	Santa Cruz Biotechnology	sc-99076	FL-137	IB & IHC
TAZ	Novus Biologicals	NB 600-220		IB & IHC
TAZ	BD Biosciences	560235	M2-616	IHC
TAZ/YAP	Cell Signaling Technology	#8418	D24E4	IB

ChIP, chromatin immunoprecipitation; FC, Flow cytometry; IB, Immunoblot; IF, Immunofluorescence; IHC, Immunohistochemistry; IP, Immunoprecipitation

Supplemental Table S2. Used primer sequences

Primers for gene expression analysis		
gene	Direction	sequences
β-actin	forward	5'-agagggaaatcgtgcgtgac-3'
	reverse	5'-caatagtgatgacctggccgt-3'
CAT	forward	5'-tcagccctggagcacagcgtcc-3'
	reverse	5'-cacgcctgtaacatgtgtttcca-3'
GSTA	forward	5'-ggagattgatgggatgaagc-3'
	reverse	5'-aacaccttttcaaaggcagg-3'
HO-1	forward	5'-taaagcgtctccacgaggtg-3'
	reverse	5'-tgttcctctgtcagcatcacc-3'
IL-6	forward	5'-gaggataccactcccaacaga-3'
	reverse	5'-aagtgcatcatcgttgttcataca-3'
MCP1	forward	5'-cttctgggcctgctgttca-3'
	reverse	5'-ccagcctactcattgggatca-3'
MIP1α	forward	5'-tgtttgctgccaagtagccac-3'
	reverse	5'-aacagtgtgaacaactgggag-3'
SRX	forward	5'-agtgcagagcctggtgg-3'
	reverse	5'-tgcagctgctggtaggctg-3'
TAZ	forward	5′-gtcaccaacagtagctcagatc-3′
	reverse	5′-agtgattacagccaggttagaaag-3′
TNFα	forward	5′-catcttctcaaattcgagtgacaa-3′
	reverse	5′-tgggagtagacaaggtacaaccc-3′
Biotin-labelled primers for DNA pulldown assay		
SRX-ARE wt	top	5'-biotin-tgtctcactctgacctagctg-3'
	bottom	5'-gcagctaggtcagagtgagac-3'
SRX-ARE mt	top	5′-biotin-tgtctcactccagcctagctg-3′
	bottom	5′-gcagctaggctggagtgagac-3′
NQO1-ARE	top	5′-biotin-tcacagtgactcagcagaat-3′
	bottom	5′-aattctgctgagtcactgtg-3′
ChIP-qPCR primers of *srx* and *nqo1* promoter		
SRX promoter	forward	5'-tgagtcaccacgctgtgcgtc-3'
	reverse	5'-acgctgtgatcctacctgtc-3'
NQO1 promoter	forward	5'-ctaatcactaactaaaggctcagaga-3'
	reverse	5'-cacagccacataatttcaatacct-3'
